# Prevalence and Source Tracing of PFAS in Shallow Groundwater Used for Drinking Water in Wisconsin, USA

**DOI:** 10.1021/acs.est.3c02826

**Published:** 2023-11-02

**Authors:** Matthew Silver, William Phelps, Kevin Masarik, Kyle Burke, Chen Zhang, Alex Schwartz, Miaoyan Wang, Amy L. Nitka, Jordan Schutz, Tom Trainor, John W. Washington, Bruce D. Rheineck

**Affiliations:** † Bureau of Drinking Water and Groundwater—Groundwater Section, Wisconsin Department of Natural Resources, Madison, Wisconsin 53707, United States; ‡ Center for Watershed Science and Education, College of Natural Resources, University of Wisconsin—Stevens Point, Stevens Point, Wisconsin 54481, United States; § Environmental Health Division—Organics, Wisconsin State Laboratory of Hygiene, Madison, Wisconsin 53707, United States; ∥ Department of Statistics, University of Wisconsin—Madison, Madison, Wisconsin 53707, United States; ⊥ Bureau of Environmental Analysis and Sustainability − Laboratory Certification, Wisconsin Department of Natural Resources, Green Bay, Wisconsin 54313, United States; # Center for Environmental Measurement and Modeling, U.S. Environmental Protection Agency, Athens, Georgia 30605, United States

**Keywords:** PFAS occurrence, emerging contaminants, human waste sources, septic system effluent, waste land application, agricultural sources, source water protection

## Abstract

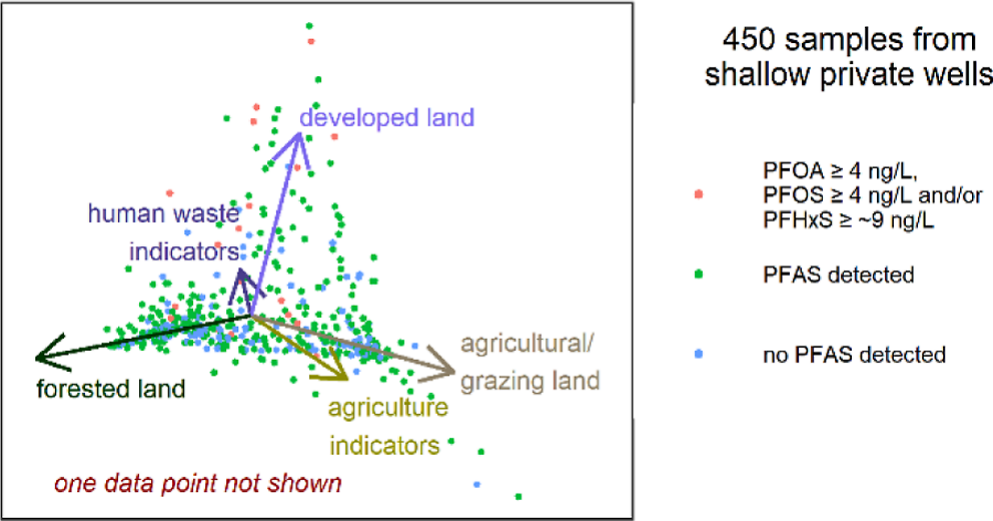

Samples from 450 homes with shallow private wells throughout the state of Wisconsin (USA) were collected and analyzed for 44 individual per- and polyfluoroalkyl substances (PFAS), general water quality parameters, and indicators of human waste as well as agricultural influence. At least one PFAS was detected in 71% of the study samples, and 22 of the 44 PFAS analytes were detected in one or more samples. Levels of PFOA and/or PFOS exceeded the proposed Maximum Contaminant Levels of 4 ng/L, put forward by the U.S. Environmental Protection Agency (EPA) in March 2023, in 17 of the 450 samples, with two additional samples containing PFHxS ≳ 9 ng/L (the EPA-proposed hazard index reference value). Those samples above the referenced PFAS levels tend to be associated with developed land and human waste indicators (artificial sweeteners and pharmaceuticals), which can be released to groundwater via septic systems. For a few samples with levels of PFOA, PFOS, and/or PFHxS > 40 ng/L, application of wastes to agricultural land is a possible source. Overall, the study suggests that human waste sources, septic systems in particular, are important sources of perfluoroalkyl acids, especially ones with ≤8 perfluorinated carbons, in shallow groundwater.

## Abstract 2

In this study of shallow groundwater, PFAS were detected in 71% of water samples from homes with private wells (4% exceeding the EPA March 2023 proposed MCLs), with indications that human waste sources are important contributors of PFAS to groundwater.

## 1 Introduction

Per- and polyfluoroalkyl substances (PFAS) are a large group of synthetic chemicals used in consumer, firefighting, and industrial products since the 1950s that pose a threat to drinking water supplies. In the past decade, environmental occurrence studies have found that PFAS occur ubiquitously in many environmental media, including treated wastewater,^1,2^ surface water,^3,4^ soil,^5,6^ and precipitation.^7−9^ In previous site- or region-specific investigations, PFAS have been found in groundwater, with concentrations varying over several orders of magnitude.^10^ Groundwater is the source of about 39% of the water supplied by public water systems in the United States as well as the source of water for private wells, which are used by about 15% of the population.^11^ Based on results from the U.S. Environmental Protection Agency’s (EPA) Third Unregulated Contaminant Monitoring Rule (UCMR3) sampling of municipal water systems conducted in 2013–2015, it was estimated that drinking water supplies exceed the 2016 EPA Health Advisory Level of 70 ng/L PFOA + PFOS for ∼6 million U.S. residents.^12^ Incorporation of more recent data indicates that PFOA + PFOS in U.S. drinking water may exceed 1 ng/L for more than 200 million people in the United States.^13^ In a recent survey of select groundwater aquifers used as a source of drinking water in the eastern United States, one or more PFAS were detected in 47% of 254 samples.^14^ In March 2023, the EPA proposed^15^ maximum contaminant levels (MCLs) of 4 ng/L for PFOA and 4 ng/L for PFOS, as well as a hazard index MCL goal that includes four additional PFAS.

Considering the importance of groundwater to drinking water supplies, more remains to be learned about the prevalence of PFAS, where they are found, and contributions from different sources. There are numerous potential types of sources of PFAS in groundwater. The source type that has received perhaps the most attention is aqueous film-forming foams (AFFFs), which are designed to be used on flammable liquid fires. AFFF discharges in training exercises and fire response are known to be a means of contaminating groundwater.^16−19^ Additionally, the presence of military sites in watersheds used for groundwater-sourced water supplies was found to increase the likelihood of municipal drinking water contamination.^12^ AFFFs, landfills,^20^ and industrial activities^12^ (direct discharges) are among the most notable potential point sources of PFAS to groundwater. Potential nonpoint sources include precipitation (PFAS have been found in Wisconsin precipitation in the single digits of ng/L^9^) as well as PFAS-containing materials that can be land-applied, such as sewage sludge (“biosolids”) from wastewater treatment plants,^12,21−23^ septage (liquid and solid waste from septic systems, holding tanks, and/or portable restrooms), industrial wastes from the manufacture of consumer products, manure,^21^ and pesticides.^24,25^ While we are not aware of any studies documenting PFAS in septage, PFAS have been found in a variety of toilet paper products.^26^ PFAS have also been found in household consumer products such as impregnation agents, paper, leather products, carpets, and other textiles and clothing.^27−31^ Due to the common presence of these product types in households, another possible source of PFAS in groundwater is septic system (onsite wastewater treatment system) effluent. Two previous studies performed in areas with many private wells and septic systems found indications that septic systems may be a source of PFAS in groundwater.^32,33^ Once released on or near the land surface, PFAS are known to accumulate at air–water interfaces,^34,35^ which are abundant in unsaturated zone soil, leading to an observed tendency to find higher concentrations at shallower subsurface depths.^16,36,37^ Other factors that can potentially influence the occurrence and transport of PFAS in the subsurface include sorption,^38,39^ generally with higher sorption of perfluoroalkyl sulfonic acids (PFSAs) compared to perfluoroalkyl carboxylic acids (PFCAs),^40,41^ and the tendency for more sorption with longer perfluoroalkyl carbon chain length.^41,42^

In this study, we characterized the prevalence of PFAS in Wisconsin’s ambient shallow groundwater using an Equal Area Grid^43,44^ approach that included collection of 450 water samples from residences with private wells. “Ambient” refers to locations at least three miles away from sites related to previously known PFAS releases where regulatory actions are pending or have already been taken. For the purposes of this study, “shallow” groundwater is considered to be groundwater from the uppermost 40 feet of the uppermost continuous local aquifer. The sample size of 450 was chosen in consideration of previous surveys of Wisconsin groundwater for agricultural chemicals,^45^ which utilized about 400 samples statewide as the minimum number needed to statistically characterize groundwater impacts. Our study is similar to that of McMahon et al.^14^ in use of a suite of water quality parameters in addition to PFAS and an Equal Area Grid approach to well selection but also differs in a few ways, most notably well type and depth. McMahon et al.^14^ sampled networks that are mostly (64%) public wells, while we sampled only shallow (as defined above) private wells.

The objective of this study was to provide a snapshot in time of the current prevalence of PFAS in shallow groundwater across Wisconsin and to better understand the levels and major source types of PFAS in groundwater, which is the source of drinking water for approximately 70% of Wisconsin residents (public and private wells combined). Sampling shallow private wells allows characterization of the quality of drinking water currently being consumed by many private well owners and is analogous to the roughly 10,000 small public systems’ wells in the state, while also enabling an improved assessment of the susceptibility of deeper groundwater supplies (which are utilized by many larger public water systems and deeper private wells) to contamination. We also utilize land use data to make inferences on the contributions of potential types of sources of PFAS to groundwater and identify risk factors for higher concentrations of PFAS in shallow private wells. To aid in identification of dispersed human waste sources in this study, water samples collected from homes with private wells were analyzed for two indicator suites, based on Nitka et al.:^46^ artificial sweeteners (acesulfame and sucralose) and pharmaceuticals (carbamazepine and sulfamethoxazole) as human waste indicators (HWIs) and metabolites of two commonly used herbicides (alachlor and metolachlor) as indicators of agricultural activities.

## 2 Materials and Methods

### 2.1 Sample Point Selection

**Figure 1:**
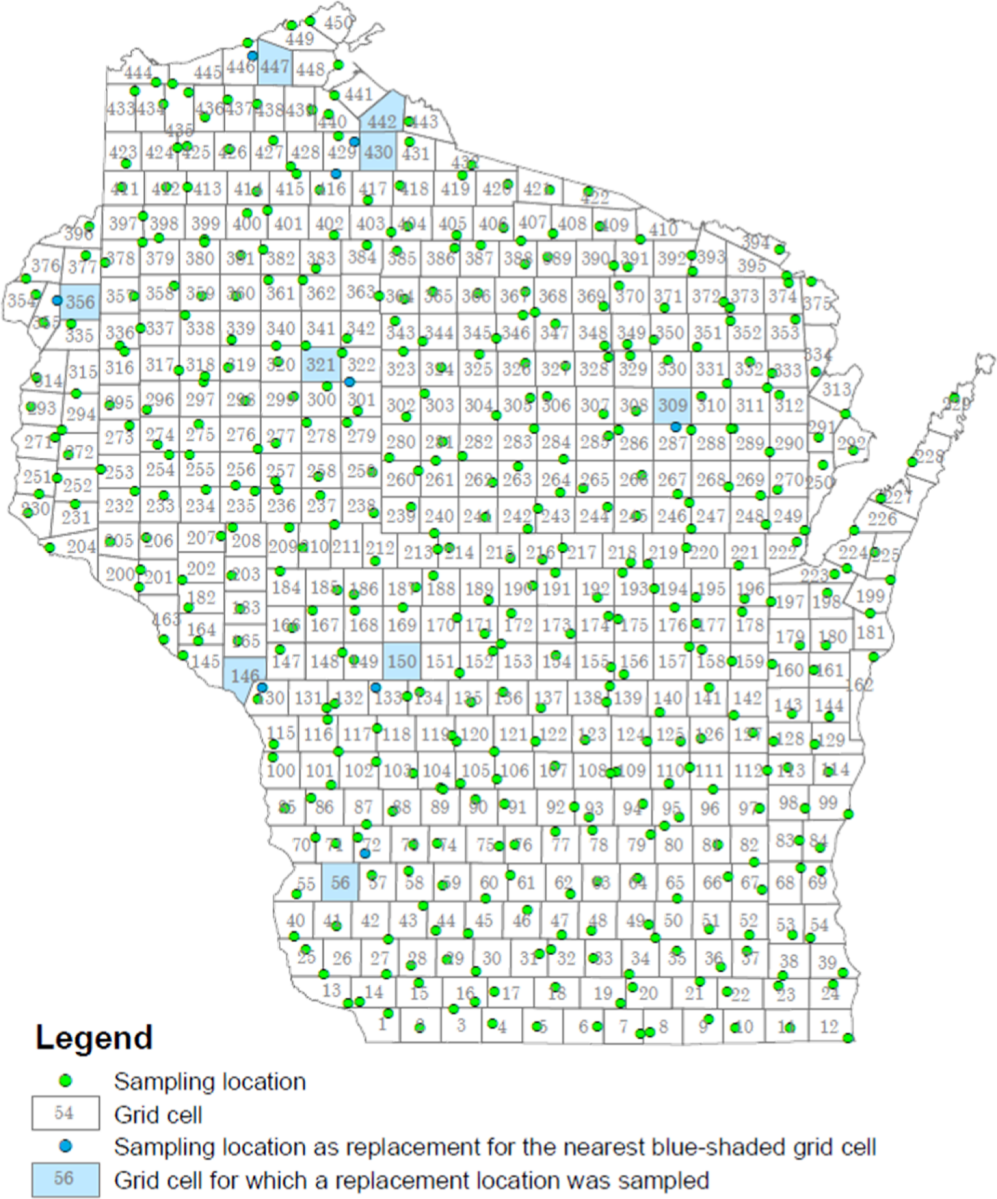
Grid cells and project sampling locations. Reproduced with permission from the Wisconsin Department of Natural Resources.

Study sampling locations were selected in three steps. In Step 1, based on the Equal Area Grid methodology,^44^ Wisconsin was divided into 450 grid cells of equal area, with a target area of 321 km^2^ (124 square miles) (Figure 1). In Step 2, lists of private water supply wells in each of the 450 grid cells meeting the following criteria were compiled: (1) a Well Construction Report (WCR) is available, and (2) casing extends downward at least to the static water level noted on the WCR but no deeper than 40 ft below the static water level. The purpose of these criteria was to sample water from homes with wells drawing relatively shallow groundwater and casing that prevents contributions from the vadose zone, enabling comparability of results across the state. In order to investigate PFAS in ambient groundwater, WCRs for locations within three miles of an existing Wisconsin Department of Natural Resources (DNR) site with actionable PFAS concentrations (as of April 22, 2022) were excluded. A large enough radius to provide an amount of safety for avoiding wells affected by a source subject to regulatory action was desired, but with typical grid cell widths around 11.1 miles, excluding areas larger than a circle with a three-mile radius would have risked excluding too many wells within a single grid cell. Additional details, including recruitment of participants with candidate private wells in Step 3, are described in the Supporting Information.

### 2.2 Sampling

Water samples were collected from a home plumbing system tap connected to a home’s water supply well. Before sampling, information provided by the homeowner/resident(s) about the presence of any treatment system was reviewed. The default sampling location was an outdoor faucet; however, if the homeowner/resident(s) indicated treatment was installed, adjustments (e.g., different tap or temporarily bypassing the treatment system) were made as necessary to sample untreated water.

Sampling was performed by trained teams of two samplers, following the procedure detailed in Section S1.1 of the Supporting Information. Briefly, water was purged until temperature, conductivity, and pH stabilized. After stabilization, water flow was turned down, then the primary sampler put on nitrile gloves, filled two 250 mL polypropylene (PP) bottles, and collected a PFAS field blank at every site by pouring laboratory-provided water into an empty 250 mL PP bottle. Following the collection of PFAS samples, additional sample bottles for non-PFAS laboratory analysis were filled. Further details on sample bottles and field QC samples are provided in Supporting Information.

### 2.3 PFAS Lab Analysis

PFAS standards were purchased from Wellington Laboratories and Cambridge Isotope Laboratories. Analytes and extracted internal standards are listed in Tables S1 and S2. Blank water (18.2 MΩ·cm resistivity) with no detectable PFAS levels was generated by an ELGA water purification system.

Extraction and analysis for PFAS (see Table 1 for a list of analytes detected and the Supporting Information for analytes without any detections) were conducted at the Wisconsin State Laboratory of Hygiene based on the ISO 21675^47^ draft method. Aqueous samples were extracted using WAX SPE cartridges and analyzed using high-performance liquid chromatography tandem mass spectrometry (HPLC-MS/MS) in negative ion mode. Analyte concentrations were quantified using isotope dilution for those analytes with an existing commercially available exact isotopically labeled standard. Analytes for which there was no commercially available exact isotopically labeled standard were quantified using extracted internal standards (isotopically labeled) of chemically similar compounds, close in retention time to the native analyte. For compounds with commercially available qualitative or quantitative standards containing branched and linear isomers, the PFAS analyte was reported as a single analyte consisting of the total amount of linear and branched isomers. Additional method details are provided in the Supporting Information.

**Table 1:** Analytes Detected in This Study and Method Detection Limits

analyte name (acid form)	analyte acronym	CAS number	detection limit (ng/L)
6:2 fluorotelomer sulfonic acid	6:2FTS	27619-97-2	0.257
perfluorobutanoic acid	PFBA	375-22-4	0.327
perfluoropentanoic acid	PFPeA	2706-90-3	0.142
perfluorohexanoic acid	PFHxA	307-24-4	0.193
perfluoroheptanoic acid	PFHpA	375-85-9	0.142
perfluorooctanoic acid	PFOA	335-67-1	0.102
perfluorononanoic acid	PFNA	375-95-1	0.140
perfluorodecanoic acid	PFDA	335-76-2	0.154
perfluoroundecanoic acid	PFUnA	2058-94-8	0.210
perfluorododecanoic acid	PFDoA	307-55-1	0.256
perfluorotridecanoic acid	PFTrDA	72629-94-8	0.183
perfluorotetradecanoic acid	PFTeDA	376-06-7	0.166
perfluoropropanesulfonic acid	PFPrS	423-41-6	0.244
perfluorobutanesulfonamide	PFBSA	30334-69-1	0.409
perfluorobutanesulfonic acid	PFBS	375-73-5	0.219
perfluoropentansulfonic acid	PFPeS	2706-91-4	0.129
perfluorohexanesulfonic acid	PFHxS	355-46-4	0.134
perfluoroheptanesulfonic acid	PFHpS	375-92-8	0.180
perfluorooctanesulfonic acid	PFOS	1763-23-1	0.135
perfluorooctanesulfonamide	PFOSA	754-91-6	0.147
*N*-ethyl perfluorooctane sulfonamidoacetic acid	NEtFOSAA	2991-50-6	0.201
perfluoro(perfluoroethyl) cyclohexanesulfonic acid	PFECHS	133201-07-7	0.181

### 2.4 Non-PFAS Lab Analysis

Compounds indicative of human waste and agricultural impacts were analyzed to aid in contaminant source tracing. The following four HWIs were analyzed: acesulfame (artificial sweetener), sucralose (artificial sweetener), carbamazepine (antiepileptic), and sulfamethoxazole (human antibiotic pharmaceutical). Additionally, four chloroacetanilide metabolites (CAAMs), alachlor ethanesulfonic acid (ESA), alachlor oxanilic acid (OA), metolachlor ESA, and metolachlor OA (for structures, see Supporting Information, Figure S1), were chosen as indicators of agricultural impacts. The parent compounds (alachlor and metolachlor) have commonly been used on corn and soybeans in Wisconsin^45,48^ (corn and soybeans are the largest crops in Wisconsin by harvested acreage^49^). The metabolites are more polar and therefore more likely to be detected in groundwater than their respective parent compounds.^50^

HWIs and CAAMs were analyzed at the University of Wisconsin–Stevens Point’s Water and Environmental Analysis Laboratory (WEAL). Site samples and quality control samples were prepared for analysis using solid-phase extraction. Extracts were analyzed using liquid chromatography tandem mass spectrometry with an electrospray ionization source (LC-ESI/MS/MS). Analysis for HWIs was adapted from EPA Method 1694 and Nitka et al. (2019). CAAM analysis was adapted from Zimmerman et al.^51^ Methods and standards are detailed in the Supporting Information.

### 2.5 Statistical and Spatial Data Analysis

Statistical analysis was performed using R for Windows version 4.3.0 (The R Foundation for Statistical Computing). Box-and-whisker plots were generated directly from all detected concentrations. For representing study sites by land use type, land use for circular areas around wells was compiled from Wiscland2,^52^ utilizing a 500 m radius, which was validated by a USGS study^53^ that evaluated different sizes and geometries for approximating the capture zone to a well. After the compilation of land use for each well, proportionality tests were then used to compare PFAS detection rates across land use categories. Specifically, for those proportionality tests, we first classified each sample as either PFAS detected (at any concentration) or no PFAS detections and then used the R function “prop.test”, which calculates *p*-values based on the chi-squared statistics under the null hypothesis of equal PFAS detection rates. Next, differences in PFAS concentrations grouped by land use type were tested using two-sample Mann-Whitney-Wilcoxon nonparametric rank sum tests after first recensoring the sum of detected PFAS by replacing any values (detected or not) below the highest detection limit of any PFAS analyte (0.409 ng/L) with 1/10th of that value (adapted from Section 5.1 of Statistical Methods in Water Resources^54^). Finally, for the generation of Spearman correlation coefficients and principal component analysis, results below the highest detection limit for each compound were recensored to 1/10th of the compound-specific detection limit (Table 1). A matrix of Spearman correlation coefficients was produced using the R function “cor” and corrected for family-wise significance following Holm’s sequential procedure.^55^ Principal component analysis was performed using the R function “prcomp”, including scaling of all variables before generating the principal components.

## 3 Results and Discussion

### 3.1 Occurrence

**Figure 2:**
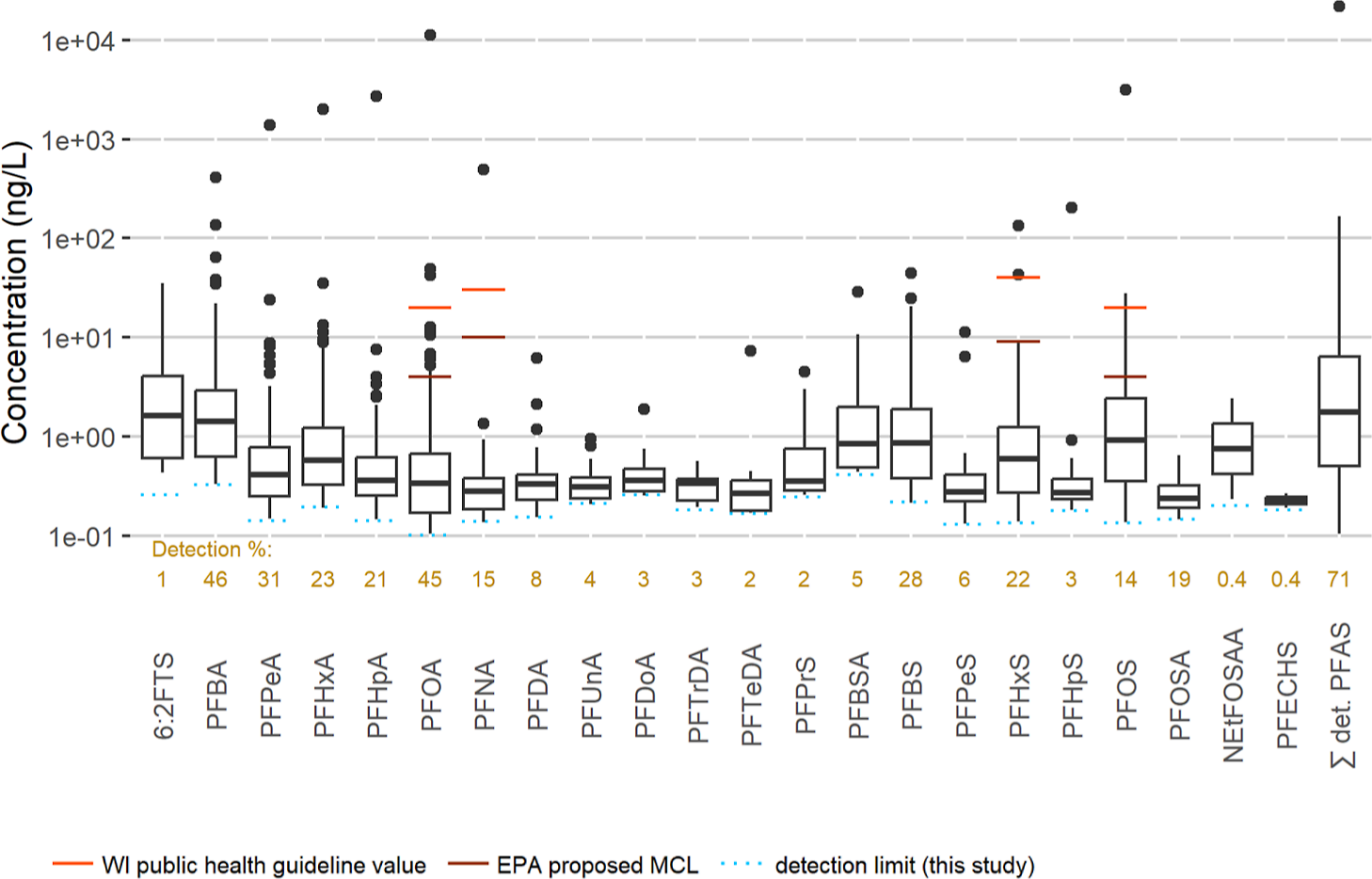
Prevalence of PFAS detected in the 450 samples (for acronyms, see Table 1). The percentage of samples with detections is shown above the compound names, while the boxes, whiskers, and points display the detected concentrations. Boxes show the 25th through 75th percentile concentrations, while whiskers (lines no greater than 1.5 times the length from the 25th to 75th percentile) and black dots combined show detected concentrations outside of the 25th through 75th percentiles. The ∑ det. PFAS variable is the sum of any PFAS that were detected in each sample.

At least one PFAS was detected in 71% of the 450 samples (Figure 2). PFBA was the most frequently detected compound (46% of samples), followed by PFOA (45% of samples). Since there is a higher detection limit for PFBA than PFOA (Table 1), the relative prevalence of PFBA in groundwater may be much higher than PFOA (discussed further in the next paragraph). For PFOA, three sample results are above the 2019 Wisconsin Department of Health Services public health value of 20 ng/L and 13 are above the March 2023 EPA proposed MCL of 4 ng/L. For PFOS, two are above the Wisconsin public health value of 20 ng/L and 11 are above the EPA proposed MCL of 4 ng/L. PFHxS results for two samples are above both the Wisconsin public health value and the EPA proposed MCL Health Based Water Concentration (40 and 9 ng/L, respectively). Overall, four of the 450 study samples (1%) had one or more PFAS above a Wisconsin public health value, and 19 of the 450 (4%) had one or more PFAS above an EPA proposed MCL (including Health Based Water Concentrations). The 71% rate of detection of one or more PFAS in this study, in which we exclusively sampled wells with relatively shallow casing, is higher than the 45% rate suggested by modeling in a recent U.S.-wide study with a different set of sample types.^56^

Because detection limits vary by analyte (Table 1), some compounds in Figure 2 are censored at higher levels than others. To compare all compounds starting from the same minimum level, Figure S2 presents boxplots with a minimum comparison level for all compounds at 0.181 ng/L, corresponding to the median detection limit of all 22 detected compounds. For three compounds, concentrations modeled below the compound-specific detection limit using regression on order statistics^57,58^ (ROS) are included. ROS was performed for all detected compounds, with only PFBA, PFDoA, and PFBS having modeled concentrations (plots in Figure S3) at or above 0.181 ng/L. At least one PFAS was detected at ≥0.181 ng/L in 65% of the study samples. Among PFCAs, inferred prevalence peaks at C4 (PFBA, also the most prevalent PFAS overall) and decreases with each increase in chain length through C7 (PFHpA, detected at ≥0.18 ng/L in 19% of the study samples). Prevalence rises again at C8 (PFOA, detected at ≥0.18 ng/L in 32% of the study samples) and then also decreases with increasing chain length through C14 (PFTeDA, detected at ≥0.18 ng/L in 2% of the study samples). For PFSAs, (inferred) detection frequencies are higher for even chain lengths, and among even chain lengths, detected or modeled prevalence ≥0.181 ng/L decreases with chain length: PFBS 29%, PFHxS 20%, and PFOS 12%. ROS modeling was performed for these comparisons of prevalence only; ROS-modeled concentrations are not used further.

While the analyte list consists of 16 precursors of perfluoroalkyl acids (PFAAs, which are combinations of the PFCA and PFSA groups), only four precursors were detected: 6:2FTS, a precursor of PFHxA and other short-chain PFCAs; PFBSA, a precursor of PFBS; PFOSA, a precursor of PFOS; and NEtFOSAA, also a precursor of PFOS. Boxplots of non-PFAS analytes/parameters are shown in Figures S4 and S5.

While the study sampling locations were selected to be representative of ambient groundwater, one sample had PFOA detected at 11,300 ng/L and eight other PFAS detected at concentrations above 100 ng/L. This occurred despite a sampling location selection process that excluded areas within three miles of a previously known site with actionable PFAS concentrations. In order to have actionable PFAS concentrations, compelling evidence of a local discharge is necessary. The fact that these levels were found, despite our site selection process avoiding previously known contamination, points to the possibility that other locations in Wisconsin with PFAS discharges capable of creating a hazard to public health may exist but had not yet been identified and/or subjected to regulatory actions at the time of the study. Additional sampling, driven by the need to protect public health, of water from other homes with private wells in a three-mile radius from the original study site (i.e., the one with the PFOA result of 11,300 ng/L) has found at least 31 additional locations with PFOA above 1000 ng/L (all samples were analyzed by Wisconsin DNR-certified laboratories using EPA Method 537.1 or a laboratory-specific isotope dilution method that meets Wisconsin DNR PFAS method expectations^59^). This site is discussed further at the end of Section 3.2.

### 3.2 Source Tracing

PFAS occurrence in groundwater may be affected by many factors. For lower concentrations, this includes the ubiquitous detection of PFAS in precipitation.^7−9^ There are 57 samples from this study (Figure S6) for which all detected PFAS were at or below the highest site median from a Wisconsin precipitation study^9^ with sample collection (91 samples from eight sites mostly reflecting ambient conditions) during 2020. Detected PFAS in the 57 samples (this study) were the C4–C9 PFCAs, PFTeDA, PFHxS, PFOS, and PFOSA. Identification of these PFAS and their levels in our study samples serves as an estimate of which PFAS in groundwater could have come from precipitation plus dry deposition, without elevated levels from discrete sources. Uncertainties in this estimate include that PFAS may accumulate in soils before breakthrough to groundwater^5,60^ and that historical levels in precipitation are largely unknown.

**Figure 3:**
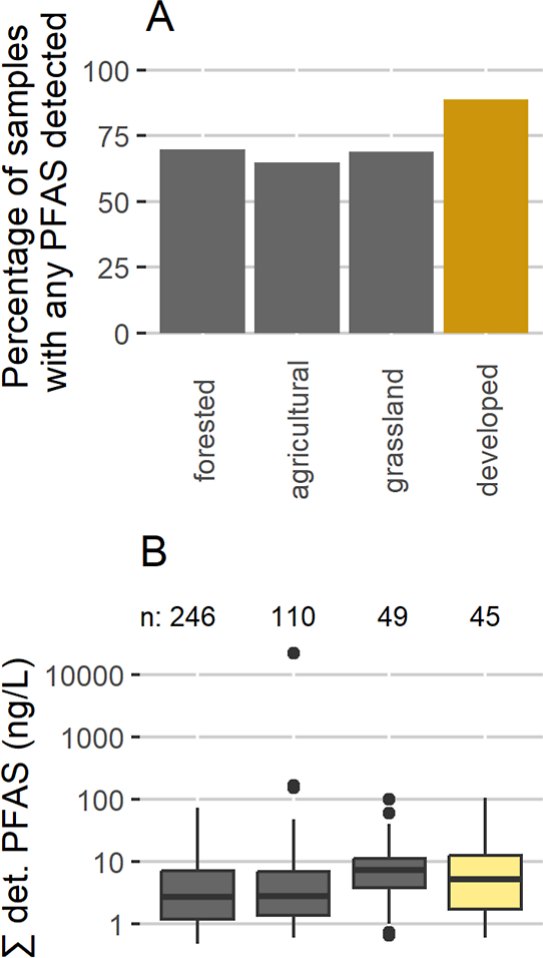
(A) Rates of PFAS detection by the largest land use type within 500 m of each sample location (see B for number of samples per category) and (B) boxplots of the sum of detected PFAS, where one or more was detected.

Differences in land use could affect PFAS occurrence at any concentration. Figure 3 shows prevalence comparisons by land use (Wiscland2^52^), with samples categorized by the highest percentage of land use in the 500 m circle around each well. Figure 3a displays PFAS detection rates across these categories, with the highest detection rate in the developed category. Proportionality tests show significant differences between developed areas (reflecting housing density) vs other categories (developed vs forested, *p* = 0.02; developed vs agricultural, *p* = 0.004; developed vs grassland, *p* = 0.04), suggesting that PFAS detections are more likely to occur in developed areas. Compound-specific detection frequencies (Figure S7) show that developed vs forested and developed vs agricultural differences are driven largely by detection rates of the C4–C7 PFCAs, PFBSA, and the C4–C8 PFSAs (except for PFHpS). Figure 3b shows, for samples with detection of one or more PFAS, box-and-whisker plots of the sum of all detected PFAS in each sample across the land use categories (for detection rate, see Figure 3a). The median values of ∑ det. PFAS are slightly higher in developed land than both forested and agricultural areas. To incorporate the magnitude of detected concentrations into two-sample Mann-Whitney-Wilcoxon nonparametric rank sum tests, data were prepared as described in Section 2.5. These rank-sum tests indicate that PFAS levels in areas with “developed” as the largest land use are significantly different from levels in both the forested (*p* = 7 × 10^–6^) and agricultural (*p* = 4 × 10^–4^) categories (comparison of developed to grassland gives an approximate—due to ties in rank—*p*-value of 0.02). These tests indicate that both detection rate and ∑ det. PFAS levels are higher in developed areas. However, it is also noteworthy that four of the five highest ∑ det. PFAS levels are in areas with either agriculture or grassland as the main land use. Land application of wastes is a possible source of PFAS in groundwater.^22,61^ While ∼18% of the study sampling sites in both the forested and developed categories had land application of wastes in proximity to the sampling site, that percentage is 37% for grassland (including land used for livestock forage production and grazing; see Supporting Information, Section S1.5, for more information) and 49% for agricultural land.

**Figure 4:**
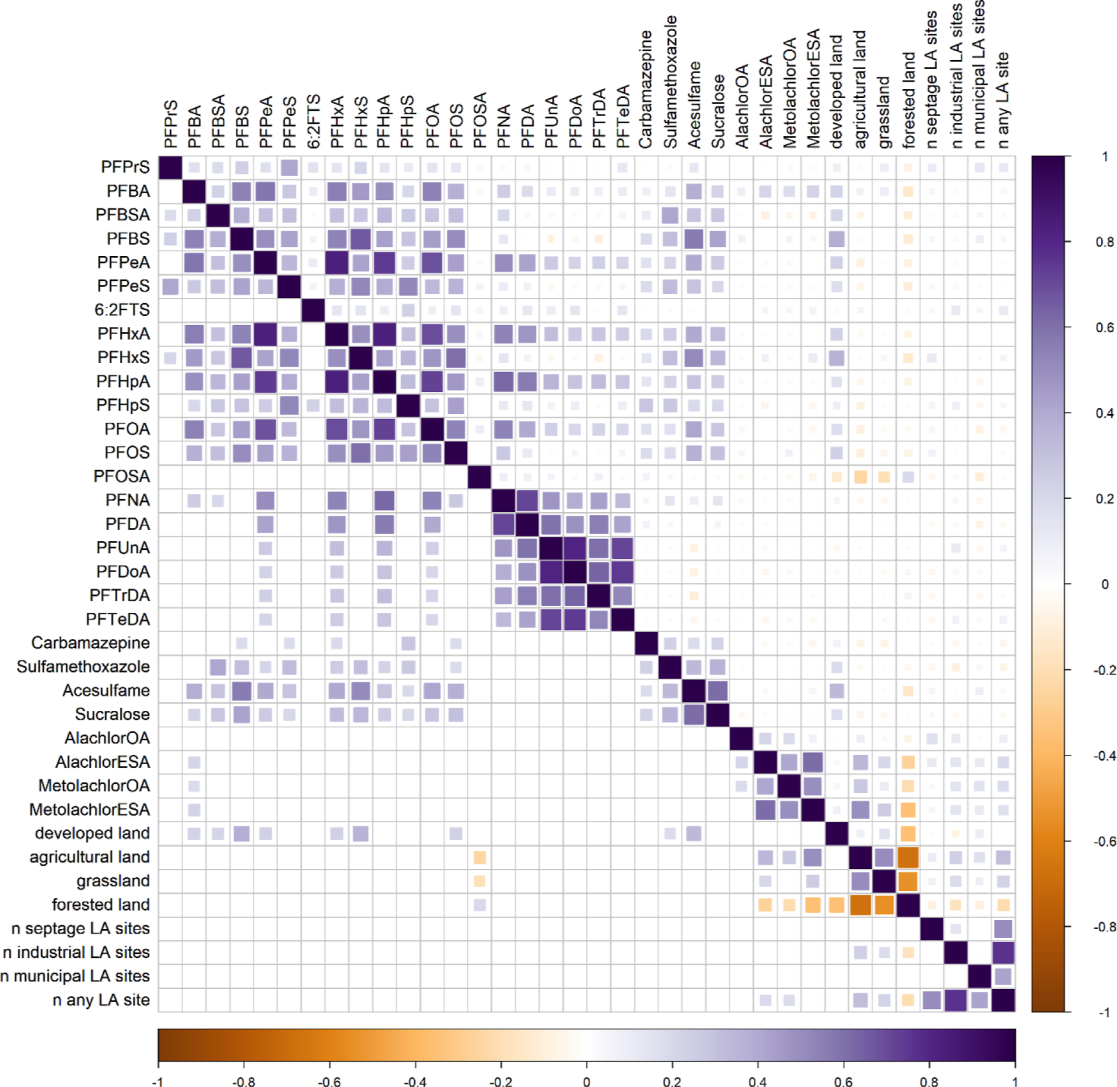
Correlation plot with intensity and amount of color indicating the value of the Spearman correlation coefficient (ρ) between variable pairs. The upper-right triangle shows all correlation values, regardless of *p*-values, while the lower-left triangle shows colored squares only for significant correlations (Holm sequentially adjusted *p* < 0.05).

Figure 4 shows Spearman ρ correlations between many study variables (additional variables are shown in Figure S8), including land use and the number of nearby waste land application sites (radius of 1000 m, reflecting the 500 m radius used for land use plus a buffer for waste application point location inaccuracies). Colors and square sizes in Figure 4 reflect the magnitude of the Spearman ρ value. Because Figure 4 depicts 630 comparisons, a small fraction of the “significant” results in this large number of comparisons could arise from chance alone. Thus, while the upper-right triangle of Figure 4 shows all Spearman ρ correlations, the lower-left triangle of Figure 4 only shows correlations that are family-wise significant at α = 0.05 after Holm sequential adjustment^55^ (for those Holm sequentially adjusted p-values, see Table S24). Where multiple contiguous PFAS homologues load significantly on a single independent variable, this outcome provides more robust evidence of a causal factor. In contrast, lone “orphan” significant comparisons may, in some cases, arise by chance. Among PFAS, a grouping of shorter chain compounds (from PFPrS through PFOS, with the exception of 6:2FTS) mostly shows significant positive correlations with each other. For example, PFHxA shows strong (Spearman ρ > 0.49) correlations with PFBA, PFBS, PFPeA, PFHxS, PFHpA, PFOA, PFOS, and PFNA (*p*-values for the named are all lower than 10^–25^). PFOA shows moderate (Spearman ρ > 0.19) to strong correlations with all other PFCAs except PFTeDA, with Spearman ρ values ranging from 0.19 (PFTeDA, *p* = 5 × 10^–3^) to 0.72 (PFHpA, *p* = 2 × 10^–71^). One notable pair is PFOA and PFNA (Spearman ρ = 0.53, *p* = 2 × 10^–31^), both of which can be a product of 8:2 FTOH degradation in the atmosphere.^62^ Combined with degradation studies,^63−66^ this suggests the possibility of a common fluorotelomer origin for those compounds. Among C10–C14 PFCAs, correlations to C8 and shorter chained PFSAs and PFBSA are sparse.

The compounds PFBS and PFBSA show moderate to strong correlations with the HWIs acesulfame, sucralose, and sulfamethoxazole (lowest Spearman ρ is PFBSA to sucralose at 0.28, *p* = 7 × 10^–7^). PFOSA is the C8 homologue of PFBSA (C4) and PFOSA is known to transform in the environment to PFOS,^2^ making PFBSA a suspected PFBS precursor. Previous studies have shown that legacy pre-2002 electrochemical fluorination AFFFs contain PFBS (and possible PFBS precursors),^17,18,67,68^ while one commercial product that has been found to contain PFBSA is “Scotchgard” produced after 2002^69^ (the latter could result in the presence of PFBSA in clothing and other household sources). In two studies of precipitation,^8,9^ PFBS was analyzed but not detected in any samples. Both legacy (produced before ∼2002) AFFFs and post-2002 Scotchgard product are possible sources of PFBS and PFBSA in shallow groundwater.^67^ The significantly higher (see Table S24 for *p*-values) prevalence of PFBS and PFBSA in samples from the developed land use category (Figure S7) and the correlations with acesulfame, sucralose, and sulfamethoxazole point toward human waste sources (septic system effluent; land application of septage or biosolids) as important ones to account for PFBS and PFBSA in shallow groundwater.

In the order the PFAS are listed in Figure 4, the compounds PFBA through PFOS (with the exceptions of 6:2FTS overall and sulfamethoxazole to PFBA and PFOA) show significant correlations with the HWIs sulfamethoxazole, acesulfame, and sucralose (among those pairs, the least significant correlation is for PFOS-sulfamethoxazole at *p* = 4 × 10^–2^). Significant correlations with developed land (represented as a decimal for the portion of land use in 500 m around the well) are present for several of those same PFAS (specifically, PFBA, PFBSA, PFBS, PFPeA, PFHxA, PFHxS, and PFOS), with the lowest Spearman ρ among these pairs being with PFBSA, with Spearman ρ = 0.21 (*p* = 2 × 10^–3^). The positive correlations between these PFAS and HWIs are an indication that human waste sources may play an important role in PFAS occurrence. This is similar to the findings of Schaider et al. that suggested that septic systems are a likely source of PFAS in private wells.^32^ Our study, however, includes a wide variety of land uses and activities, such as land application of biosolids and septage, which may also be PFAS sources. The presence of PFAS in biosolids, at varying concentrations, is well documented;^21,22,70^ however, we are not aware of any studies documenting PFAS levels in septage.

The HWIs chosen for this study can be useful in identifying influence from human waste sources, especially in agricultural areas where contributions from land-applied wastes are minimal.^46^ However, in addition to samples from septic systems,^71,72^ the HWIs have also been found in biosolids,^73,74^ making them nonunique tracers among dispersed human waste sources. While HWIs can be present in landfills^75^ (another dispersed source of human wastes), acesulfame and sucralose were approved by the U.S. Food and Drug Administration between 1988 and 1999,^76^ making it unlikely that they are associated with historic landfill sources. The solid–liquid partitioning behavior of HWIs in wastewater treatment (including septic systems) may reflect where the most mass loading to the environment occurs. On the one hand, acesulfame and sucralose are highly water-soluble^77^ and have been found in wastewater effluent at levels ∼50 times higher than sludge.^77,78^ On the other hand, sulfamethoxazole and carbamazepine have activated sludge sorption coefficients (*K*_d_) of 9.7 and 91 L/kg, respectively,^79^ indicating strong partitioning to sludge (biosolids) rather than wastewater. Sulfamethoxazole has been detected in private wells on Cape Cod, with septic systems being a likely source.^80^ Since discharge to groundwater from a septic system can readily result in high concentrations of mobile compounds such as artificial sweeteners and many short-chain PFAAs, and also considering the higher PFAS correlations with developed land than agricultural land, septic systems can be viewed as a likely source of PFAS in groundwater. However, other human waste sources cannot be ruled out as possibly contributing some of the PFAS to groundwater.

PFBA was detected at significantly higher concentrations in an area of western Wisconsin (Figure S9) compared to the rest of the state (two-sample nonparametric rank sum test *p* = 8 × 10^–12^). The water solubility of PFBA has been estimated to be 2 orders of magnitude higher than that of PFOA^81^ and higher sorption of PFOA compared to PFBA has been found,^38,82^ suggesting relatively high mobility of PFBA in groundwater. While definitive attribution of the source(s) and transport mechanism(s) of the higher PFBA levels in western Wisconsin shallow groundwater is beyond the scope of this study, it is noteworthy that PFBA has been found as the predominant PFAS compound in some impacted environmental waters to the west in Minnesota.^83,84^ High PFAS levels in soil samples in major downwind directions of factories using or manufacturing PFAS have been found in New York/Vermont^85^ and New Jersey,^86^ suggesting that aerial contamination can contaminate soil downwind of a major source. The New York/Vermont study additionally attributed groundwater PFOA contamination to that pathway.^85^

Of the CAAM analytes, three are significantly correlated with PFBA, but no other significant relationships are found between CAAMs and PFAS (Figure 4). PFBA was among the most frequently detected PFAS in Wisconsin precipitation^9^ and is the most frequently detected PFAS in this study (Figure 2). While there are a variety of possible sources of PFAS in agriculture,^87^ there are no significant positive correlations between PFAS analytes and agricultural land use (Figure 4). The lack of significant correlations between the CAAMs and PFAS, other than three CAAMs with PFBA (discussed further below), suggests a limited relationship between CAAMs and PFAS. PFBA in western Wisconsin (Figure S9) could be from a nonagricultural source (e.g., precipitation, possibly with higher historical PFAS levels), and this raises the question of how much those western Wisconsin samples influence the correlations of PFBA with the three CAAMs showing significant correlations (Figure 4). With the 40 western Wisconsin (Figure S9) samples removed, the Spearman ρ correlation of PFBA with the three CAAMs with which PFBA has significant correlations (*p* < 0.05) decreases as follows: 0.22 to 0.13 for alachlor ESA, 0.22 to 0.17 for metolachlor ESA, and 0.19 to 0.18 for metolachlor OA. These comparisons are another indication, to the extent that it is viewed as likely that the higher PFBA in the area of western Wisconsin is attributable to something other than agriculture, of a limited role of agricultural land use on overall PFAS occurrence.

**Figure 5:**
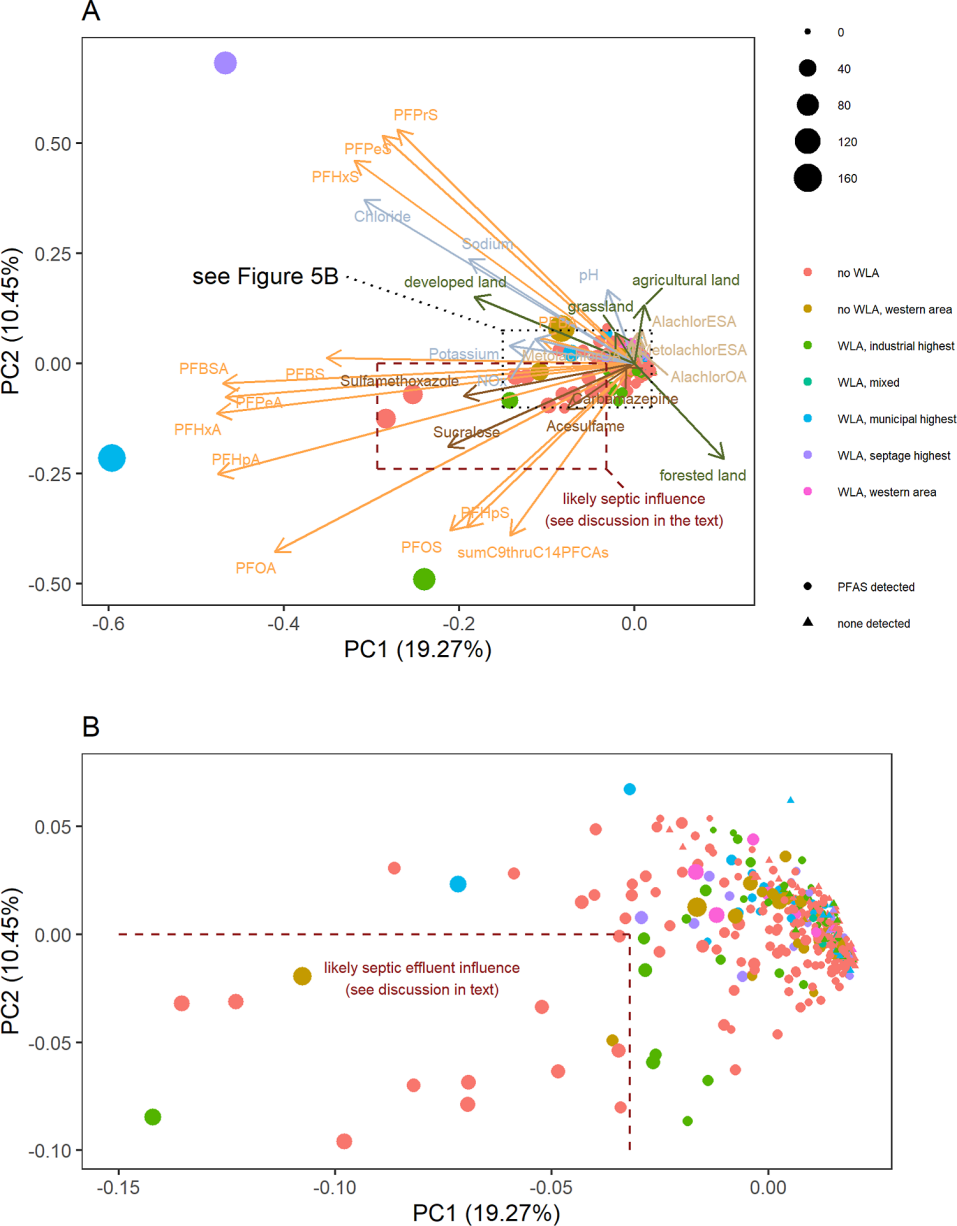
Principal component analysis plots for all samples (A) and zoomed in on a smaller area (B), which is indicated by the block dotted rectangle on (A). Symbol shape indicates whether PFAS were detected, and symbol size is proportional to the sum of detected PFAS. Colors of symbols indicate if waste land application (WLA) has been permitted nearby and also indicate which samples are located in the western area with higher PFBA concentrations.

Principal component analysis (Figure 5) was performed on the results of 449 samples (the sample with PFOA at 11,300 ng/L is excluded). While waste land application is not the only diagnostic of the PFAS source for an individual sample, it can be informative to the source type where samples cluster. The area of the dark red dashed rectangle (Figure 5a) encloses the end of the loading vectors of three of the HWIs (acesulfame, sucralose, and sulfamethoxazole). Most of the samples in this region have no nearby waste land application, leaving septic system effluent as a likely source for most of these samples (and possibly also others closer to the plot origin in the same direction as those loadings). Stacked column plots showing the PFAS signatures (combinations of detected compounds) for the 19 samples with PFAS levels above the March 2023 EPA proposed MCLs are shown in Figure S10.

The loading vectors of PFPrS, PFPeS, and PFHxS, as well as those of developed land, chloride, and sodium, share a similar angular direction. A source other than septic systems (possibly legacy AFFFs) is likely for samples in that direction, though unlike within the dark red dashed box, there are few samples at moderate loading values in this angular direction. Three outliers are visible in Figure 5a. The sample toward the upper left had PFHxS detected at 42.6 ng/L. While the data point is colored in the septage land application category, there are also industrial sites in the area (in addition to the possibility of an AFFF source, although no PFOS was detected). For the outlying data points in the lower left and lower central portions of Figure 5a, no industrial sites are located nearby. Of these three outliers, the latter two (i.e., ones with negative values of PC2) can be viewed as having a higher likelihood of PFAS contamination from a waste land application source. While waste land application is a suspect for source attribution of those two outliers, the loading vectors of agricultural land and the four agricultural indicator compounds suggest that most agricultural practices are not a major source of PFAS in groundwater.

Figure 5 omits the sample with PFOA detected at 11,300 ng/L. The well for that sample is located near agricultural fields that have received biosolids, septage, and paper mill sludge. This raises the possibility of waste land application as the source. Investigation into the source(s) of the groundwater contamination in the area is part of a DNR-led site investigation that commenced in January 2023.

### 3.3 Implications for Source Water Protection

This study was done to characterize current levels of PFAS in Wisconsin’s shallow groundwater, water that approximately 70% of the state’s population currently uses as their drinking water supply. Furthermore, what is shallow groundwater today will typically move deeper over time, with the potential for PFAS to increasingly become a drinking water quality issue for municipal water systems that draw water from deeper high-capacity wells. Several lines of evidence point to human waste sources as contributors of PFAS to groundwater, with effluent discharged from septic systems likely a major source of PFAS in groundwater. Detected PFAS were generally low overall compared to the March 2023 EPA proposed MCLs, but 19 samples (4%) had levels above those of the proposed MCLs. Aside from the five samples with the highest ∑ det. PFAS, septic systems are a likely source for most of the other 15 samples above the EPA proposed MCLs. This study points to the importance of reducing PFAS in septic system wastewater streams and the need for more effective technologies and management strategies for these waste streams in order to protect drinking water supplies.

Results presented here also illustrate a different character of the PFAS problem for developed versus agricultural communities. Owners of shallow wells in developed areas can expect a greater likelihood that PFAS are present in their water supply at currently detectable levels. On the other hand, although the majority of agricultural and other lower population density locations have a lower likelihood of PFAS detection, a few samples with especially high PFAS levels were from locations with either agriculture or grassland as the highest land use. Absent characterization of wastes and utilization of that information in determining which wastes are applied to agricultural land, potable wells in agricultural settings that received land application of wastes would, from a cautious perspective, need to be regarded as having a high risk of containing especially high PFAS levels.

## Supporting Information Available

Additional details related to sampling design, laboratory materials and methods, land use data set, prevalence with a minimum comparison level and ROS-modeled concentrations, non-PFAS field and lab parameters, comparison to Wisconsin 2020 PFAS precipitation levels, individual PFAS by land use category, expanded correlation plot, PFBA map, and stacked column plots for PFAS composition of project samples above EPA March 2023 proposed MCLs (PDF)
Additional tables detailing land use around each of the study wells, field parameters, lab results for all primary samples and field quality control samples, and Holm-adjusted *p*-values for the comparisons shown on Figure 4 (XLSX)
