# Supplementary material for: Prevalence and Source Tracing of PFAS in Shallow Groundwater Used for Drinking Water in Wisconsin, USA
Source: Environ Sci Technol. 2023 Nov 2;57(45):17415–26. doi: 10.1021/acs.est.3c02826 (PMC10653221; doi:10.1021/acs.est.3c02826)
Supplement: Supplementary file 1 — es3c02826_si_001.pdf [file es3c02826_si_001.pdf]

Supporting Information for

Prevalence and source tracing of PFAS in shallow groundwater used for drinking water in Wisconsin, USA

Matthew Silver[1], William Phelps[1], Kevin Masarik[2], Kyle Burke[3], Chen Zhang[3], Alex Schwartz[3], Miaoyan Wang[4], Amy L. Nitka[2], Jordan Schutz[1], Tom Trainor[5], John W. Washington[6], Bruce D. Rheineck[1]

[1]Bureau of Drinking Water and Groundwater—Groundwater Section, Wisconsin Department of Natural Resources, Madison, Wisconsin 53707, United States

[2]Center for Watershed Science and Education, College of Natural Resources, University of Wisconsin—Stevens Point, Stevens Point, Wisconsin 54481, United States

[3]Environmental Health Division - Organics, Wisconsin State Laboratory of Hygiene, Madison, Wisconsin 53707, United States

[4]Department of Statistics, University of Wisconsin—Madison, Madison, Wisconsin 53707, United States

[5]Bureau of Environmental Analysis and Sustainability – Laboratory Certification, Wisconsin Department of Natural Resources, Green Bay, Wisconsin 54313, United States

[6]Center for Environmental Measurement and Modeling, U.S. Environmental Protection Agency, Athens, Georgia 30605, United States

The Supporting Information contains:

- 46 pages
- 24 tables (Tables S20-S24 in a separate XLSX file)
- 10 figures

Contents

1.0 Additional description of materials and methods S4
1.1 Sampling grid and sample point selection S4
1.2 Sampling procedure S4
1.3 PFAS laboratory analysis S7
1.3.1 Analytes and standards S7
1.3.2 PFAS extraction S14
1.3.3 PFAS Analysis by HPLC-MS/MS S15
1.3.4 PFAS Quality control and quantitation S16
1.4 Laboratory analysis of human waste indicators and herbicide metabolites S25
1.4.1 Purchased chemicals S27
1.4.2 Aqueous extraction method for human waste indicators S28
1.4.3 Aqueous extraction method for chloroacetanilide metabolites S28
1.4.4 LC/MS/MS method for human waste indicators S28
1.4.5 LC/MS/MS method for chloroacetanilide metabolites S29
1.4.6 Quality control – non-PFAS organics S29
1.5 Inorganics and total organic carbon S30
1.6 Land use data analysis S34
2.0 Results and discussion S35
2.1 Occurrence S35
2.2 Source tracing S39
References S46

Figures

Figure S1. Structures from EPA CompTox7 of the human waste indicator (HWI) and chloroacetanilide metabolite (CAAM) analytes S26

Figure S2. Boxplots of detected and modeled PFAS concentrations at or above 0.181 ng/L S36

Figure S3. Regression on order statistics models for the three compounds with modeled concentrations above 0.181 ng/L S37

Figure S4. Prevalence of non-PFAS analytes/parameters in the 450 samples S38

Figure S5. Field measurement values of water pH, temperature (◦C) and conductivity (µS/cm) in the 450 samples S39

Figure S6. Project samples with all detected PFAS below 2020 Wisconsin precipitation levels S39

Figure S7. Rate of detection of individual PFAS, at any concentration, across land use categories S41

Figure S8. Correlation plot with intensity and amount of color indicating the value of the Spearman correlation coefficient (ρ) between two variables S43

Figure S9. A) Concentrations of PFBA at the 450 project sites and B) comparison of concentrations in the Western Area (shaded yellow on A) vs. the rest of the state S44

Figure S10. Stacked column plots of the PFAS detected in 18 of the 19 samples with one or more PFAS above the EPA March 2023 proposed MCLs S45

1.0 Additional description of materials and methods

1.1 Sampling grid and sample point selection

The range in grid cell areas (Figure 1 of the manuscript) is 316 to 326 km^2 (122-126 square miles).

Step 3 of sampling location selection consisted of several sub-steps and iterations. Initially, five WCRs were selected at random off of each list from Step 2. Invitation letters, offering the opportunity to participate in the study, were sent to, typically, three addresses from the five selected (based on determination of a useable mailing address). If more than one positive response to the mailed invitation was received for a grid cell, the participant selected for sampling was chosen randomly. If only one positive response was received, that location was selected for sampling. If no positive responses were received for a grid cell, letters were sent to additional randomly selected participants in the same grid cell (from the list produced in Step 2), in three additional mailing rounds until a positive response was received. Grid cells for the study and sampling locations are shown in Figure 1 of the manuscript. For nine out of the 450 grid cells, a second sampling location in a nearby grid cell was used, as no invited participant in the original (blue-shaded) grid cell accepted the study participation invitation.

1.2 Sampling procedure

Sampling was performed in teams of two trained samplers. Sampling kits were prepared before leaving for the field. After collection, samples were stored in battery/electrical-powered coolers capable of achieving cooling down to -20 °C, with the option for also setting cooling temperatures within the range 0°C to 6°C. An overview of sample bottles, field QC samples, preservation and holding times is provided in Table S1.

Due to potential for sample contamination, in particular for PFAS, the following precautions were taken:

1. Samplers typically wore cotton clothing that had been washed at least three times prior to sampling.
2. No personal care products were applied after leaving the vehicle to begin sampling.
3. Personal care products such as sunscreen and mosquito repellant were chosen in consideration of product information in PFAS Sampling Guidance from the Michigan Department of Environmental Quality[1]. These products were only applied in the morning and during breaks.
4. No consumption of food or drink during sampling, with the exception of bottled water.
5. Hands were washed thoroughly before filling sample bottles and care was taken to touch only parts of the bottles and caps that do not come in contact with sample water.

After arriving at a site, contact was made with the homeowner/resident if present and an information packet was left for them. Both samplers worked on measurement of field parameters. When it was time to fill sample bottles, sampling was performed following the “clean hands/dirty hands” technique. One member of the sampling team was designated prior to arrival at the site as "dirty hands" while the second member was designated as "clean hands". All operations involving contact with the sample bottle were handled by the individual designated as "clean hands”, while the sampler designated as “dirty hands” performed other tasks (e.g., labeling, note taking, and turning the faucet on before sampling).

Steps performed were as follows:

1. Identification of the tap to be sampled. Typically, an outdoor tap was sampled. In some cases, an indoor tap near the pressure tank, before any installed treatment system, was sampled.
2. Run water, purging about 10 gallons through a hose directed to an area away from the home or other buildings.
3. Begin measuring field parameters using a flow-thru cell or, if not available, with probes at the bottom of a 5-gallon bucket.
4. Continue until stabilization of water temperature, specific conductance and pH, then those parameters plus dissolved oxygen were recorded.
5. Sampler 1 (designated to keep clean hands) opened the PFAS sampling kit (LDPE bag with a loosely tied knot) and put on the powderless nitrile gloves.
6. Both PFAS sample bottles were filled so that they were about 90% full, allowing room for expansion when the sample freezes, then the field blank was collected by pouring lab-supplied blank water in one 250 mL PP bottle into another.
7. Remaining sample bottles were filled in this order: 1000 mL amber glass bottle (human waste and herbicide indicators), 250 mL amber glass pre-filled with sulfuric acid (NOx, TAN and chloride), 250 mL HDPE (metallic and other elements), 60 mL HDPE (conductivity, pH, alkalinity).
8. The second sampler began labeling sample bottles with a fine-tip permanent marker after all sample bottles for PFAS, including the field blank, had been filled and capped.
9. Sample bottles were placed in cold storage following Table S1.

Table S1. Sample bottles, field QC samples, preservation and holding times

Analytical group	Primary sample	Field Reagent Blanks	Field Duplicates	Matrix spike (MS) Matrix spike duplicate (MSD)	Preservation	Holding time
PFAS	250 mL PP bottle, as labeled in Appendix B	1 per sample	1 collected for every sample, 1 per batch analyzed	None since method uses isotope dilution	Frozen at -20°Cto <0°C	90 days to extraction
CAAMs and PPCPs	1000 mL amber glass bottle	1 for every 20 samples	1 for every 20 samples	1 MS/MSD for every 20 samples; 1000 mL is sufficient to do MS/MSD.	Refrigeration at 0°C to 6°C (not frozen)	28 days to extraction
Inorganics Bottle 1: metallic and other elements by ICP-OES	60 mL HDPE bottle, unpreserved until arrival in laboratory (HNO_3 added in lab)	6 total	6 total	Independent from field QC samples, one MS every 10 field samples, MSD as needed to assess instrument reproducibility; 60 mL sample volume is sufficient for MS/MSD and the sample.	0°C to 6°C (not frozen)	28 days
Inorganics Bottle 2: NOx, TAN and chloride by FIA, and TOC	250 mL amber glass bottle, containing H_2SO_4 as preservative	6 total	6 total	Independent from field QC samples, one MS every 20 field samples for all; one MSD included for TAN every 20 samples.	0°C to 6°C (not frozen), H_2SO_4 added to bottles before sampling	28 days

Analytical group	Primary sample	Field Reagent Blanks	Field Duplicates	Matrix spike (MS) Matrix spike duplicate (MSD)	Preservation	Holding time
Inorganics Bottle 3: alkalinity and conductivity	250 mL HDPE bottle, unpreserved	6 total	6 total	None	0°C to 6°C (not frozen) (HNO_3 added in the lab after sampling)	14 days

Notes:

1. PPCPs = Pharmaceuticals, personal care products, artificial sweeteners
2. CAAMs = Chloroacetanilide herbicide metabolites
3. HDPE = high density polyethylene; PP = polypropylene
4. NOx = nitrate plus nitrite nitrogen; TAN = total ammonia/ammonium nitrogen, TOC = total organic carbon (non-purgeable)

1.3 PFAS laboratory analysis

PFAS laboratory analysis was performed at the Wisconsin State Laboratory of Hygiene.

1.3.1 Analytes and standards

Analytes and standards are listed in Table S2. Methanol (HPLC grade), and ammonium acetate (MS grade) were purchased from Honeywell and Sigma Aldrich, respectively. Table S3 shows additional information on analytical standards. For certain compounds, multiple names/abbreviations are shown in the tables due to inconsistencies in those identifiers between various relevant documentation (i.e., analytical standard product catalogues and other published methods cited in Table S4).

Table S2. Project analyte list and analytical standard source

Product Code	Vendor	Compound acronym	Full Name
EPA-537PDS-R1 Native PFAS Mix	Wellington	PFHxA	Perfluoro-n-hexanoic acid
		PFHpA	Perfluoro-n-heptanoic acid
		PFOA	Perfluoro-n-octanoic acid
		PFNA	Perfluoro-n-nonanoic acid
		PFDA	Perfluoro-n-decanoic acid
		PFUnA	Perfluoro-n-undecanoic acid
		PFDoA	Perfluoro-n-dodecanoic acid
		PFTrDA	Perfluoro-n-tridecanoic acid
		PFTeDA	Perfluoro-n-tetradecanoic acid
		HFPO-DA	2,3,3,3-Tetrafluoro-2-(1,1,2,2,3,3-heptafluoropropoxy)-propanoic acid
		N-MeFOSAA	N-methyl perfluorooctanesulfonamidoacetic acid
		N-EtFOSAA	N-ethyl perfluorooctanesulfonamidoacetic acid
		PFBS	Perfluoro-1-butanesulfonic acid
		PFHxS	Perfluoro-1-hexanesulfonic acid
		PFOS	Perfluoro-1-octanesulfonic acid
		DONA	Dodecafluoro-3H-4,8-dioxanonanoic acid
		9Cl-PF3ONS	9-Chlorohexadecafluoro-3-oxanone-1-sulfonic acid
		11Cl-PF3OUdS	11-Chloroeicosafluoro-3-oxaundecane-1-sulfonic acid
PFBA	Wellington	PFBA	Perfluoro-n-butanoic acid
PFPeA	Wellington	PFPeA	Perfluoro-n-pentanoic acid
L-PFPeS	Wellington	PFPeS	Perfluoro-1-pentanesulfononic acid
L-PFHpS	Wellington	PFHpS	Perfluoro-1-heptanesulfonic acid
L-PFNS	Wellington	PFNS	Perfluoro-1-nonanesulfonic acid
L-PFDS	Wellington	PFDS	Perfluoro-1-decanesulfonic acid

Product Code	Vendor	Compound acronym	Full Name
L-PFDoS	Wellington	PFDoS	Perfluoro-1-dodecanesulfonic acid
4:2 FTSA	Wellington	4:2 FTSA	1H,1H,2H,2H-Perfluorohexane sulphonic acid
6:2 FTS	Cambridge Isotope	6:2 FTSA	1H,1H,2H,2H-Tridecafluorooctane-1-sulphonic acid
8:2 FTS	Cambridge Isotope	8:2 FTSA	1H,1H,2H,2H-Perfluorodecanesulphonic acid
10:2 FTS	Wellington	10:2 FTSA	1H,1H,2H,2H-Perfluorododecane sulfonic acid
FOSA-I	Wellington	PFOSA	Perfluorooctanesulphonamide
N-MeFOSA	Cambridge Isotope	N-MeFOSA	N-Methyl Perfluorooctanesulfonamide
N-EtFOSA	Cambridge Isotope	N-EtFOSA	N-Ethyl Perfluorooctanesulfonamide
N-MeFOSE	Cambridge Isotope	N-MeFOSE	2-(N-methylperfluoro-1-octanesulfonamido)-ethanol
N-EtFOSE	Cambridge Isotope	N-EtFOSE	2-(N-ethylperfluoro-1-octanesulfonamido)-ethanol
PFECHS	Wellington	PFECHS	Perfluoro-4-ethylcyclohexanesulfonic acid
L-PFPrS	Wellington	PFPrS	Perfluoro-1-propanesulfonic acid
PFBSA	Cambridge Isotope	PFBSA	Perfluorobutanesulfonamide
PFHxSA	Cambridge Isotope	PFHxSA	Perfluorohexanesulfonamide
FPrPA	Wellington	FPrPA/3:3 FTCA	3-Perfluoropropyl propanoic acid / 3:3 fluorotelomer carboxylic acid
FPePA	Wellington	FPePA/5:3 FTCA	3-Perfluoropentyl propanoic acid / 5:3 fluorotelomer carboxylic acid
FHpPA	Wellington	FHpPA/7:3 FTCA	3-Perfluoroheptyl propanoic acid / 7:3 fluorotelomer carboxylic acid

Product Code	Vendor	Compound acronym	Full Name
FHUEA	Wellington	FHUEA/6:2 FTUCA	2H-Perfluoro-2-octenoic acid / 6:2 fluorotelomer unsaturated carboxylic acid
FOUEA	Wellington	FOUEA/8:2 FTUCA	2H-Perfluoro-2-decenoic acid / 8:2 fluorotelomer unsaturated carboxylic acid
FDUEA	Wellington	FDUEA/10:2 FTUCA	2H-Perfluoro-2-dodecenoic acid / 10:2 fluorotelomer unsaturated carboxylic acid

Table S3. Project extracted internal standard list and analytical standard source

Product Code	Vendor	Mass-labeled compound acronym
MPFAC-24ES Mass Labelled PFAS Mix	Wellington	$^{13}C_4$-PFBA
		$^{13}C_5$-PFPeA
		$^{13}C_5$-PFHxA
		$^{13}C_4$-PFHpA
		$^{13}C_8$-PFOA
		$^{13}C_9$-PFNA
		$^{13}C_6$-PFDA
		$^{13}C_7$-PFUnA
		$^{13}C_2$-PFDoA
		$^{13}C_2$-PFTeDA
		$^{13}C_8$-PFOSA
		d_3-N-MeFOSAA
		d_5-N-EtFOSAA
		$^{13}C_3$-PFBS
		$^{13}C_3$-PFHxS
		$^{13}C_8$-PFOS
		$^{13}C_2$-4:2FTSA
		$^{13}C_2$-6:2FTSA

Product Code	Vendor	Mass-labeled compound acronym
		$^{13}C_2$-8:2FTSA
M3HFPO-DA	Wellington	$^{13}C_3$-HFPO-DA
d3-N-MeFOSA-M	Wellington	d_3-N-MeFOSA
d5-N-EtFOSA-M	Wellington	d_5-N-EtFOSA
d7-N-MeFOSE-M	Wellington	d_7-N-MeFOSE
d9-N-EtFOSE-M	Wellington	d_9-N-EtFOSE
MFHUEA	Wellington	$^{13}C_2$-FHUEA
MFOUEA	Wellington	$^{13}C_2$-FOUEA
MFDUEA	Wellington	$^{13}C_2$-FDUEA
13C2, D4, 10:2 FTS	Cambridge Isotope	$^{13}C_2D_4$-10:2 FTSA

The rationale for the choice of extracted internal standards is provided in Table S4. For PFECHS, the extracted internal standard in OTM-45[2] is $^{18}O_2$-PFHxS. During method development, PFECHS was quantitated with both $^{13}C_8$-PFOS and $^{13}C_3$-PFHxS. Recoveries using $^{13}C_3$-PFHxS were equal or better when compared to recoveries using $^{13}C_8$-PFOS. Since recoveries using $^{13}C_3$-PFHxS were equal or better than using $^{13}C_8$-PFOS and OTM-45 also uses labeled PFHxS (just a different isotope), $^{13}C_3$-PFHxS was chosen as the extracted internal standard. The extracted internal standard recovery was used to adjust target analyte concentrations.

Table S4. PFAS analytes, WI public health values and extracted internal standards. Classes of PFAS within the analyte list are shown in italics on rows preceding each group.

Acronym	CAS number	2019/2020 Wisconsin public health value (ng L^{-1})	Mass-labeled compound acronym	Mass-labeled compound acronym reference/ rationale
Perfluoroalkyl carboxylic acids (terminal compounds in the environment)				
PFBA	375-22-4	10,000	$^{13}C_4$-PFBA	EPA Draft Method 1633
PFPeA	2706-90-3		$^{13}C_5$-PFPeA	EPA Draft Method 1633

Acronym	CAS number	2019/2020 Wisconsin public health value (ng L^{-1})	Mass-labeled compound acronym	Mass-labeled compound acronym reference/ rationale
PFHxA	307-24-4	150,000	$^{13}C_5$-PFHxA	EPA Draft Method 1633
PFHpA	375-85-9		$^{13}C_4$-PFHpA	EPA Draft Method 1633
PFOA	335-67-1	20[1]	$^{13}C_8$-PFOA	EPA Draft Method 1633
PFNA	375-95-1	30	$^{13}C_9$-PFNA	EPA Draft Method 1633
PFDA	335-76-2	300	$^{13}C_6$-PFDA	EPA Draft Method 1633
PFUnA	2058-94-8	3,000	$^{13}C_7$-PFUnA	EPA Draft Method 1633
PFDoA	307-55-1	500	$^{13}C_2$-PFDoA	EPA Draft Method 1633
PFTrDA	72629-94-8		$^{13}C_2$-PFDoA	EPA Draft Method 1633 uses the average of $^{13}C_2$-PFTeDA and $^{13}C_2$-PFDoA
PFTeDA	376-06-7	10,000	$^{13}C_2$-PFTeDA	EPA Draft Method 1633
Perfluoroalkyl sulfonic acids (terminal compounds in the environment)				
PFPrS	423-41-6		$^{13}C_3$-PFBS	Barzen-Hanson and Field (2015)
PFBS	375-73-5	450,000	$^{13}C_3$-PFBS	EPA Draft Method 1633
PFPeS	2706-91-4		$^{13}C_3$-PFHxS	EPA Draft Method 1633
PFHxS	355-46-4	40	$^{13}C_3$-PFHxS	EPA Draft Method 1633
PFHpS	375-92-8		$^{13}C_8$-PFOS	EPA Draft Method 1633
PFOS	1763-23-1	20[1]	$^{13}C_8$-PFOS	EPA Draft Method 1633
PFNS	68259-12-1		$^{13}C_8$-PFOS	EPA Draft Method 1633
PFDS	335-77-3		$^{13}C_8$-PFOS	EPA Draft Method 1633
PFDoS	79780-39-5		$^{13}C_8$-PFOS	EPA Draft Method 1633
n:2 fluorotelomer sulfonic acids (precursors in the environment of other PFAS including PFCAs of varying chain lengths)				
4:2 FTSA	757124-72-4		$^{13}C_2$-4:2FTS	EPA Draft Method 1633
6:2 FTSA	27619-97-2		^{13}C2-6:2FTS	EPA Draft Method 1633

Acronym	CAS number	2019/2020 Wisconsin public health value (ng L^{-1})	Mass-labeled compound acronym	Mass-labeled compound acronym reference/ rationale
8:2 FTSA	39108-34-4		^{13}C2-8:2FTS	EPA Draft Method 1633
10:2 FTSA	120226-60-0		$^{13}C_2$-10:2FTS	Exact isotope label
Fluorotelomer n:3 saturated (likely terminal compounds in the environment) and n:2 unsaturated (precursors/environmental intermediates) carboxylic acids				
3:3 FTCA	356-02-5		$^{13}C_4$-PFPeA	EPA Draft Method 1633
5:3 FTCA	914637-49-3		$^{13}C_5$-PFHxA	EPA Draft Method 1633
7:3 FTCA	812-70-4		$^{13}C_5$-PFHxA	EPA Draft Method 1633
6:2 FTUCA	70887-86-6		$^{13}C_2$-6:2FTUCA	Exact isotope label; OTM 45
8:2 FTUCA	70887-84-2		$^{13}C_2$-8:2FTUCA	Exact isotope label; OTM 45
10:2 FTUCA	70887-94-4		$^{13}C_2$-10:2FTUCA	Exact isotope label
Fluorosulfonamide/sulfonamido substances (PFBSA and PFHxSA are likely precursors in the environment of PFBS and PFHxS, respectively; all others are precursors of PFOS)				
PFBSA	30334-69-1		$^{13}C_3$-PFHxS	Meng et al. (2022)
PFHxSA	41997-13-1		$^{13}C_8$-PFOS	Meng et al. (2022)
PFOSA	754-91-6	20[1]	$^{13}C_8$-PFOSA	EPA Draft Method 1633
N-MeFOSA	31506-32-8		D3-NMeFOSA	EPA Draft Method 1633
N-MeFOSE	24448-09-7		D7-NMeFOSE	EPA Draft Method 1633
N-EtFOSA	4151-50-2	20[1]	D5-NEtFOSA	EPA Draft Method 1633
N-EtFOSE	1691-99-2	20[1]	D9-NEtFOSE	EPA Draft Method 1633
N-MeFOSAA	2355-31-9		D3-NMeFOSAA	EPA Draft Method 1633
N-EtFOSAA	2991-50-6	20[1]	D5-N-EtFOSAA	EPA Draft Method 1633
Ether-containing fluorosubstances				
HFPO-DA	13252-13-6	300	$^{13}C_3$-HFPO-DA	EPA Draft Method 1633
DONA	919005-14-4	3,000	$^{13}C_4$-PFHpA	EPA Method 533
9Cl-PF3ONS	756426-58-1		$^{13}C_8$-PFOS	EPA Method 533
11Cl-PF3OUdS	763051-92-9		$^{13}C_8$-PFOS	EPA Method 533

Acronym	CAS number	2019/2020 Wisconsin public health value (ng L^{-1})	Mass-labeled compound acronym	Mass-labeled compound acronym reference/ rationale
Cyclic analogue of PFOS				
PFECHS	133201-07-7		$^{13}C_3$-PFHxS	See text

Notes (excluding isotope superscripts):

1) Superscript[1]: Wisconsin health advisory level of 20 ng L^{-1} applies to the sum of detected concentrations of PFOA, PFOS, PFOSA, NEtFOSA, NEtFOSAA and NEtFOSE.
2) In the reference list at the end of the document: OTM 45[2], EPA Draft Method 1633[3], Barzen-Hanson and Field (2015)[4], Meng et al. (2022)[5]; EPA Method 533[6]

1.3.2 PFAS extraction

Samples were extracted with a PromoChrom automated extraction system (AES). If samples contained light particulates, a PromoChrom in-line filter was used to prevent contamination and damage to AES lines and pumps.

Internal standard mix was added to each quality control sample and field sample, in the bottle in which it was collected, and allowed to equilibrate for a minimum of fifteen minutes. AES lines were rinsed with water and methanol before each extraction.

SPE cartridges were pre-conditioned in three separate steps with 4 mL ammonium hydroxide in methanol, methanol, and water, rinsing at a flow rate of 5 mL min^{-1} for each solvent. Samples were then added to the SPE cartridges in ~4 mL increments at a flow rate of 3 mL min^{-1}. After the entire samples passed through the cartridges, 4 mL each of water and 25 mM ammonium acetate buffer were added successively at 3 mL min^{-1}. Cartridges were then blown to dryness with 4 mL of air flowing at 10 mL min^{-1} through each cartridge followed by nitrogen flow at approximately 10 L min^{-1} for 10 minutes.

At the elution step, each cartridge was rinsed with 4 mL methanol at a rate of 0.5 mL min^{-1} (the sample bottle and in-line filter are rinsed first with this methanol), followed by 4 mL 60mM ammonium hydroxide in methanol at 0.5 mL min^{-1}. Extracts were collected in 15-mL polypropylene centrifuge tubes. Extracts were further filtered through a graphitized carbon black (ENVI-Carb) cartridge immediately after the initial

SPE step. The carbon SPE cartridge was rinsed with at least 6 mL 60mM ammonium hydroxide in methanol before samples were loaded. Sample extracts were added to rinsed cartridges at about 3-5 mL min^{-1} and collected in 15-mL polypropylene centrifuge tubes.

Filtered extracts were evaporated to between 0.5-1 mL under a gentle stream of nitrogen (~0.7 L min^{-1}) in a heated water bath at 60-65°C. Sample volume was adjusted to exactly 1 mL with MeOH before extracts were filtered through a glass fiber filter or polypropylene syringe filter.

1.3.3 PFAS Analysis by HPLC-MS/MS

Analysis was performed on a Waters Acquity UPLC followed by an Applied Biosystems/SCIEX Q-Trap 5500 triple quadrupole mass spectrometer:

Analytical column: Acquity UPLC BEH C18 1.7 MM 2.1x50 mm

Isolator column: Acquity UPLC Reverse-phase BEH 2.1x50 mm

Injection volume: 2 µL

Mobile Phase: (A) 2 mM ammonium acetate in H2O:MeOH (95:5), (B) MeOH

Column temperature: 40°C

Steps - Table S5:

Step	Total Time (min)	Flow Rate (mL/min)	A (%)	B (%)
0	0.00	0.200	95	5
1	0.50	0.200	95	5
2	1	0.250	80	20
3	4	0.350	50	50
4	6	0.350	40	60
5	7	0.400	30	70
6	8.5	0.400	10	90
7	9	0.350	0	100
8	10.5	0.300	0	100
9	11.5	0.200	95	5
10	13.5	0.200	95	5

Analysis performed on a SCIEX ExionLC followed by an Applied Biosystems/SCIEX Triple Quad 7500 mass spectrometer:

Analytical column: Acquity UPLC BEH C18 1.7 MM 2.1x50 mm Column

Isolator column: Acquity UPLC Hybrid Reversed Phase 2.1x50 mm

Injection volume: 1 µL

Mobile Phase: (A) 2 mM ammonium acetate in H2O:MeOH (95:5), (B) Methanol

Column temperature: 40°C

Steps - Table S6:

Step	Time (min)	Flow (mL/min)	A (%)	B (%)
0	0.0	0.300	95	5
1	0.2	0.300	95	5
2	0.5	0.300	80	20
3	3.0	0.300	65	35
4	3.5	0.300	45	55
5	11.5	0.300	5	95
6	12.5	0.300	5	95
7	12.6	0.300	95	5
8	15.0	0.300	95	5

1.3.4 PFAS Quality control and quantitation

An initial demonstration of capability was performed, including demonstrating low LC system background calibration verification using a quality control standard (a calibration standard purchased and prepared separately from calibration solutions, which must be 70 – 130% of the true value), precision of solid-phase extraction (SPE; %RSD <30% for four replicates), accuracy of solid-phase extraction (average recovery within 65 – 135% for each analyte in four replicates), and determination of method detection limits (MDLs) (based on 40 CFR Appendix B to Part 136 guidelines) (Table S7). Ongoing quality control included analysis of method blanks (Milli-Q water, extracted by SPE), laboratory control spikes (Milli-Q water spiked with

analytes, extracted by SPE), and field duplicates, each performed for every extraction batch. Calibration checks were performed every 10 samples. Isotope dilution was performed by adding spikes of extracted internal standards in the sample bottle. Table S7 also shows the method reporting limit (MRL) for each compound. The MRL is the minimum concentration reported as a quantitative value for a method analyte in a sample following analysis. This defined concentration is no lower than the concentration of the lowest calibration standard for that analyte and is only used if the recovery in the lowest standard is within 50 – 150%. The extracted internal standard recovery was used to adjust target analyte concentrations. Calibration by isotope dilution was utilized for those PFAS with an exact mass-labeled isotope (Table S3). Calibration by internal standard was utilized for other compounds.

Table S7. Method detection limits (MDLs) and method reporting limits (MRLs). MDLs in this table are for exactly 250 mL of sample; for median detection limits based on actual sample volumes, see Table 1 of the manuscript.

Analyte	Abbreviation(s)	CAS No.	MDL (ng L^{-1})	MRL (ng L^{-1})
Perfluoro-n-butanoic acid	PFBA	375-22-4	0.346	1.00
Perfluoro-1-propanesulfonic acid	PFPrS	423-41-6	0.16	1.00
3-Perfluoropropyl propanoic acid	FprPA, 3:3 FTCA	356-02-5	0.119	1.00
Perfluoro-n-pentanoic acid	PFPeA	2706-90-3	0.15	1.00
Perfluoro-1-butanesulfononic acid	PFBS	375-73-5	0.124	1.00
1H,1H,2H,2H-Perfluorohexane sulphonic acid	4:2 FTSA	757124-72-4	0.19	1.00
Perfluoro-n-hexanoic acid	PFHxA	307-24-4	0.204	1.00
Perfluorobutane sulfonamide	PFBSA	30334-69-1	0.16	1.00
Perfluoro-1-pentanesulfononic acid	PFPeS	2706-91-4	0.136	1.00
2,3,3,3-Tetrafluoro-2-(1,1,2,2,3,3-heptafluoropropoxy)-propanoic acid	HFPO-DA	13252-13-6	0.192	1.00
Perfluoro-n-heptanoic acid	PFHpA	375-85-9	0.15	1.00
3-Perfluoropentyl propanoic acid	FpePA, 5:3 FTCA	914637-49-3	0.118	1.00
Perflioro-1-hexanesulfonic acid	PFHxS	355-46-4	0.142	1.00

Analyte	Abbreviation(s)	CAS No.	MDL (ng L^{-1})	MRL (ng L^{-1})
Dodecafluoro-3H-4,8-dioxanonanoic acid	DONA	919005-14-4	0.128	1.00
2H-Perfluoro-2-octenoic acid	FHUEA, 6:2 FTUCA	70887-86-6	0.088	1.00
Perfluoro-4-ethylcyclohexanesulfonic acid	PFECHS	133201-07-7	0.127	1.00
1H,1H,2H,2H-Tridecafluorooctane-1-sulphonic acid	6:2 FTSA	27619-97-2	0.272	1.00
Perfluoro-n-octanoic acid	PFOA	335-67-1	0.108	1.00
Perfluoro-1-heptanesulfonic acid	PFHpS	375-92-8	0.098	1.00
Perfluorohexanesulfonamide	PFHxSA	41997-13-1	0.071	1.00
Perfluoro-1-octanesulfonic acid	PFOS	1763-23-1	0.143	1.00
Perfluoro-n-nonanoic acid	PFNA	375-95-1	0.148	1.00
3-Perfluoroheptyl propanoic acid	FhpPA, 7:3 FTCA	812-70-4	0.139	1.00
2H-Perfluoro-2-decenoic acid	FOUEA, 8:2 FTUCA	70887-84-2	0.193	1.00
9-Chlorohexadecafluoro-3-oxanone-1-sulfonic acid	9Cl-PF3ONS	756426-58-1	0.182	1.00
1H,1H,2H,2H-Perfluorodecanesulphonic acid	8:2 FTSA	39108-34-4	0.262	1.00
Perfluoro-n-decanoic acid	PFDA	335-76-2	0.163	1.00
Perfluoro-1-nonanesulfonic acid	PFNS	68259-12-1	0.182	1.00
N-methyl perfluorooctanesulfonamidoacetic acid	N-MeFOSAA	2355-31-9	0.219	1.00
N-ethyl perfluorooctanesulfonamidoacetic acid	N-EtFOSAA	2991-50-6	0.212	1.00
Perfluorooctane sulfonamide	FOSA, PFOSAm	754-91-6	0.155	1.00
Perfluoro-n-undecanoic acid	PFUnA, PFUdA	2058-94-8	0.222	1.00

Analyte	Abbreviation(s)	CAS No.	MDL (ng L^{-1})	MRL (ng L^{-1})
Perfluoro-1-decanesulfonic acid	PFDS	335-77-3	0.11	1.00
2H-Perfluoro-2-dodecenoic acid	FDUEA, 10:2 FTUCA	70887-94-4	0.186	1.00
11-Chloroeicosafluoro-3-oxaundecane-1-sulfonic acid	11Cl-PF3OudS	763051-92-9	0.149	1.00
1H,1H,2H,2H-Perfluorododecane sulfonic acid	10:2 FTSA	120226-60-0	0.387	1.00
Perfluoro-n-dodecanoic acid	PFDoA	307-55-1	0.135	1.00
Perfluoro-1-dodecanesulfonic acid	PFDoS	79780-39-5	0.247	1.00
Perfluoro-n-tridecanoic acid	PFTrDA	72629-94-8	0.193	1.00
N-Methyl Perfluorooctanesulfonamide	N-MeFOSA	31506-32-8	1.00	2.00
2-(N-methylperfluoro-1-octanesulfonamido)-ethanol	N-MeFOSE	24448-09-7	0.281	1.00
N-Ethyl Perfluorooctanesulfonamide	N-EtFOSA	4151-50-2	0.694	2.00
2-(N-ethylperfluoro-1-octanesulfonamido)-ethanol	N-EtFOSE	1691-99-2	0.212	1.00
Perfluoro-n-tetradecanoic acid	PFTeDA	376-06-7	0.175	1.00

Table S8. Method blank data (n=52).

Compound Name (CAS No.)	Mean (ng L^{-1})	Max (ng L^{-1})
PFBA (375-22-4)	0.026	0.527
PFPeA (2706-90-3)	0.011	0.457
PFBS (375-73-5)	0.000	0.000
4:2 FTSA (757124-72-4)	0.000	0.000
PFHxA (307-24-4)	0.014	0.536
PFPeS (2706-91-4)	0.000	0.000
HFPO-DA (13252-13-6)	0.000	0.000
PFHpA (375-85-9)	0.009	0.282

Compound Name (CAS No.)	Mean (ng L^{-1})	Max (ng L^{-1})
PFHxS (355-46-4)	0.001	0.043
DONA (919005-14-4)	0.000	0.011
6:2 FTSA (27619-97-2)	0.031	0.933
PFOA (335-67-1)	0.044	0.587
PFHpS (375-92-8)	0.000	0.000
PFOS (1763-23-1)	0.003	0.177
PFNA (375-95-1)	0.012	0.536
9Cl-PF3ONS (756426-58-1)	0.000	0.000
8:2 FTSA (39108-34-4)	0.000	0.000
PFDA (335-76-2)	0.014	0.431
PFNS (68259-12-1)	0.000	0.000
N-MeFOSAA (2355-31-9)	0.000	0.000
N-EtFOSAA (2991-50-6)	0.000	0.000
FOSA (754-91-6)	0.004	0.115
PFUnA (2058-94-8)	0.008	0.262
PFDS (335-77-3)	0.000	0.000
11Cl-PF3OudS (763051-92-9)	0.000	0.000
PFDoA (307-55-1)	0.008	0.306
PFDoS (79780-39-5)	0.000	0.000
PFTrDA (72629-94-8)	0.001	0.030
N-MeFOSA (31506-32-8)	0.006	0.119
N-MeFOSE (24448-09-7)	0.001	0.063
N-EtFOSA (4151-50-2)	0.005	0.080
N-EtFOSE (1691-99-2)	0.006	0.275
PFTeDA (376-06-7)	0.006	0.235
10:2 FTSA (120226-60-0)	0.002	0.020
PFPrS (423-41-6)	0.000	0.000
FprPA (356-02-5)	0.000	0.000
PFBSA (30334-69-1)	0.000	0.000
FpePA (914637-49-3)	0.000	0.000

Compound Name (CAS No.)	Mean (ng L^{-1})	Max (ng L^{-1})
FHUEA (70887-88-6)	0.002	0.058
PFECHS (133201-07-7)	0.002	0.064
PFHxSA (41997-13-1)	0.000	0.000
FhpPA (812-70-4)	0.002	0.109
FOUEA (70887-84-2)	0.001	0.071
FDUEA (70887-94-4)	0.000	0.022

Table S9. Control spike data. LCS nominally spiked at 1 ng L^{-1} (2 ng L^{-1} for PFBA), LCS1 nominally spiked at 10 ng L^{-1} (20 ng L^{-1} for PFBA), and LCS2 nominally spiked at 40 ng L^{-1} (80 ng L^{-1} for PFBA). Units for all values in Table are ng L^{-1}.

	LCS (n=19)			LCS1 (n=19)			LCS2 (n=15)		
**Compound Name (CAS No.)**	**Average**	**Min**	**Max**	**Average**	**Min**	**Max**	**Average**	**Min**	**Max**
PFBA (375-22-4)	117	80	165	87	74	104	107	96	125
PFPeA (2706-90-3)	116	85	153	87	74	108	106	94	124
PFBS (375-73-5)	112	79	153	87	76	110	107	94	129
4:2 FTSA (757124-72-4)	106	73	155	81	67	96	100	87	112
PFHxA (307-24-4)	115	82	169	89	75	112	109	93	130
PFPeS (2706-91-4)	116	79	149	86	61	99	109	94	122
HFPO-DA (13252-13-6)	113	77	141	84	70	111	107	88	129
PFHpA (375-85-9)	111	69	163	85	74	102	108	93	131
PFHxS (355-46-4)	113	79	140	85	64	113	106	91	131
DONA (919005-14-4)	118	90	157	89	75	117	109	90	138
6:2 FTSA (27619-97-2)	107	73	148	83	73	101	102	89	117
PFOA (335-67-1)	120	86	171	87	73	103	106	88	128
PFHpS (375-92-8)	117	80	159	89	75	103	110	98	131
PFOS (1763-23-1)	113	80	138	86	65	114	108	97	134
PFNA (375-95-1)	115	83	157	85	73	118	106	83	136

9Cl-PF3ONS (756426-58-1)	96	67	138	78	56	93	95	71	111
8:2 FTSA (39108-34-4)	109	78	154	86	76	101	103	92	120
PFDA (335-76-2)	115	81	144	87	75	114	105	92	136
PFNS (68259-12-1)	105	81	153	82	69	103	101	90	118
N-MeFOSAA (2355-31-9)	108	68	139	88	74	109	108	95	130
N-EtFOSAA (2991-50-6)	107	73	146	86	72	107	107	92	134
FOSA (754-91-6)	109	64	164	84	72	100	107	95	121
PFUnA (2058-94-8)	108	78	152	87	68	111	106	91	134
PFDS (335-77-3)	94	65	124	76	59	95	96	81	121
11Cl-PF3OudS (763051-92-9)	97	75	123	80	64	107	99	82	133
PFDoA (307-55-1)	107	72	133	87	65	118	108	91	136
PFDoS (79780-39-5)	88	42	121	66	38	94	92	73	108
PFTrDA (72629-94-8)	98	55	149	83	67	117	102	81	121
N-MeFOSA (31506-32-8)	107	51	142	85	74	102	113	99	155
N-MeFOSE (24448-09-7)	110	59	148	82	63	96	108	95	123
N-EtFOSA (4151-50-2)	104	35	170	85	69	99	113	101	138
N-EtFOSE (1691-99-2)	104	62	155	81	64	100	103	84	122
PFTeDA (376-06-7)	111	65	148	85	69	110	108	93	135
10:2 FTSA (120226-60-0)	103	66	161	82	67	104	103	91	123
PFPrS (423-41-6)	104	78	135	79	68	103	101	86	117
FprPA (356-02-5)	105	70	131	81	66	113	103	90	131
PFBSA (30334-69-1)	138	77	177	101	68	164	127	103	174
FpePA (914637-49-3)	108	77	154	84	63	105	104	94	119
FHUEA (70887-88-6)	110	64	150	85	70	105	103	90	129
PFECHS (133201-07-7)	106	69	146	82	58	100	102	90	123
PFHxSA (41997-13-1)	129	77	184	101	68	172	129	101	173
FhpPA (812-70-4)	101	55	146	79	63	103	100	86	117
FOUEA (70887-84-2)	108	63	138	82	65	113	108	95	130
FDUEA (70887-94-4)	107	82	149	84	63	103	107	90	119

Table S10. Instrument calibration check standard recoveries (n=65). Injection of calibration standard at nominal concentration of 1 ng L^{-1} (2 ng L^{-1} for PFBA).

Compound Name (CAS No.)	Average (ng L^{-1})	Min (ng L^{-1})	Max (ng L^{-1})
PFBA (375-22-4)	96	72	111
PFPeA (2706-90-3)	98	88	120
PFBS (375-73-5)	98	86	118
4:2 FTSA (757124-72-4)	93	78	114
PFHxA (307-24-4)	98	86	117
PFPeS (2706-91-4)	98	88	120
HFPO-DA (13252-13-6)	100	83	117
PFHpA (375-85-9)	96	80	116
PFHxS (355-46-4)	98	80	119
DONA (919005-14-4)	101	72	119
6:2 FTSA (27619-97-2)	92	78	112
PFOA (335-67-1)	98	84	115
PFHpS (375-92-8)	98	87	122
PFOS (1763-23-1)	97	74	118
PFNA (375-95-1)	100	87	123
9Cl-PF3ONS (756426-58-1)	97	68	119
8:2 FTSA (39108-34-4)	97	82	127
PFDA (335-76-2)	100	79	121
PFNS (68259-12-1)	96	80	113
N-MeFOSAA (2355-31-9)	99	78	127
N-EtFOSAA (2991-50-6)	96	79	117
FOSA (754-91-6)	98	90	123
PFUnA (2058-94-8)	97	81	121
PFDS (335-77-3)	95	72	122
11Cl-PF3OudS (763051-92-9)	93	73	121
PFDoA (307-55-1)	98	76	126
PFDoS (79780-39-5)	98	76	120
PFTrDA (72629-94-8)	96	63	137

Compound Name (CAS No.)	Average (ng L^{-1})	Min (ng L^{-1})	Max (ng L^{-1})
N-MeFOSA (31506-32-8)	97	78	129
N-MeFOSE (24448-09-7)	102	76	128
N-EtFOSA (4151-50-2)	98	77	113
N-EtFOSE (1691-99-2)	100	70	124
PFTeDA (376-06-7)	98	78	126
10:2 FTSA (120226-60-0)	96	84	117
PFPrS (423-41-6)	89	76	115
FprPA (356-02-5)	96	68	122
PFBSA (30334-69-1)	105	91	128
FpePA (914637-49-3)	97	75	124
FHUEA (70887-88-6)	98	77	114
PFECHS (133201-07-7)	97	84	115
PFHxSA (41997-13-1)	98	87	118
FhpPA (812-70-4)	100	81	121
FOUEA (70887-84-2)	97	82	117
FDUEA (70887-94-4)	98	85	120

Table S11. Extracted internal standard recoveries, in percent, for the 450 samples. For samples that were injected multiple times due to low recovery in the first injection, only the higher recovery is used. Recoveries were calculated from continuing calibration verification standards.

Compound	min	mean	max	Compound	min	mean	max
PFBA	32	70	100	FOSA	32	69	97
PFPeA	32	72	104	PFUnA	31	65	107
PFBS	28	69	98	PFDS	28	67	94
4:2 FTSA	30	70	130	11Cl-PF3OudS	28	67	94
PFHxA	29	71	113	PFDoA	17	61	111
PFPeS	29	70	100	PFDoS	0	50	97
HFPO-DA	33	72	115	PFTrDA	17	61	111
PFHpA	31	72	102	N-MeFOSA	3	52	83

Compound	min	mean	max	Compound	min	mean	max
PFHxS	29	70	100	N-MeFOSE	0	54	86
DONA	31	72	102	N-EtFOSA	1	46	73
6:2 FTSA	23	71	141	N-EtFOSE	1	51	81
PFOA	20	72	103	PFTeDA	0	50	97
PFHpS	28	67	94	10:2 FTSA	23	62	96
PFOS	28	67	94	PFPrS	28	69	98
PFNA	32	71	104	FprPA	32	72	104
9Cl-PF3ONS	20	71	103	PFBSA	29	70	100
8:2 FTSA	32	69	106	FpePA	29	71	113
PFDA	33	68	100	FHUEA	35	74	117
PFNS	28	67	94	PFECHS	29	70	100
N-MeFOSAA	30	64	95	PFHxSA	28	67	94
N-EtFOSAA	29	62	90	FhpPA	29	71	113
FOSA	32	69	97	FOUEA	34	72	112
PFUnA	31	65	107	FDUEA	30	65	95

1.4 Laboratory analysis of human waste indicators and herbicide metabolites

Laboratory analysis of all compounds other than PFAS was performed at the University of Wisconsin-Stevens Point's Water and Environmental Analysis Lab. Figure S1 displays structures from EPA CompTox[7] of the human waste indicator (HWI) and chloroacetanilide metabolite (CAAM) analytes.

Acesulfame

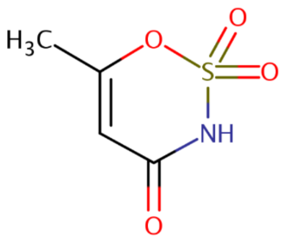

Carbamazepine

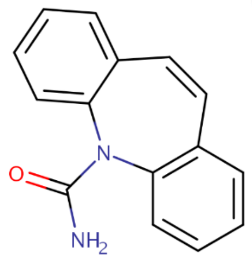

Sucralose

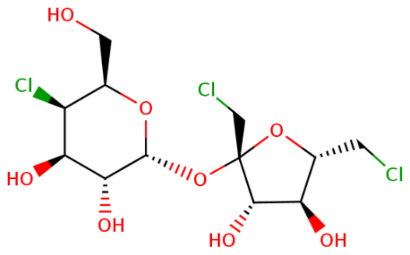

Sulfamethoxazole

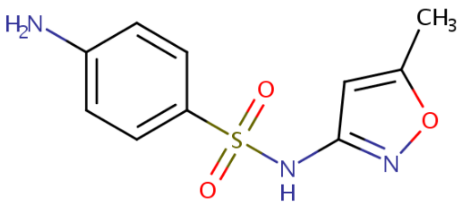

Alachlor ESA

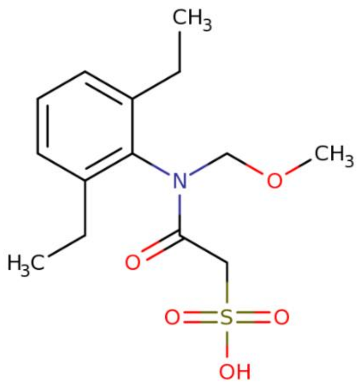

Alachlor OA

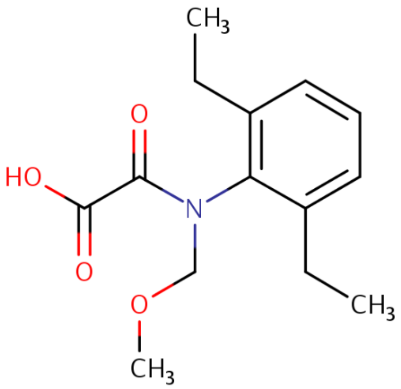

Metolachlor ESA

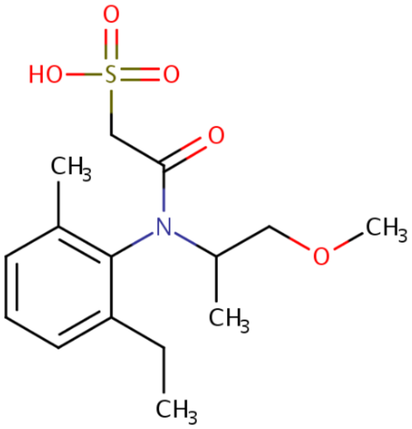

Metolachlor OA

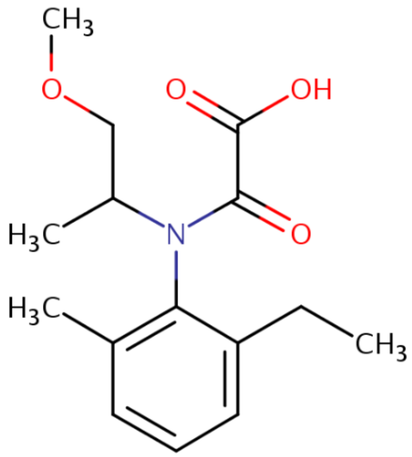

Figure S1. Structures from EPA CompTox[7] of the human waste indicator (HWI) and chloroacetanilide metabolite (CAAM) analytes.

1.4.1 Purchased chemicals

Methanol (LC/MS Optima Grade CAS 67-56-1), ethyl acetate (Optima Grade CAS 141-78-6), and ethylenediaminetetraacetic acid (EDTA) (Certified ACS CAS 6381-92-6) were purchased from Fisher Scientific. Acetic acid (ACS Reagent 16-19-7) was purchased from Sigma-Aldrich. Sulfuric acid (ACS Grade CAS 7647-01-0) was purchased from VWR. Ultrapure water (18.2 MΩ·cm) was supplied by an Elga water system. Analytical standards and sources are listed in Table S12.

Table S12. Non-PFAS organic compounds: target analytes, extracted surrogate, and injection internal standards

Compound Type	Method	Compounds	CAS Number	Source
Analytes	HWI	Acesulfame	33665-90-6	SA
		Carbamazepine	298-46-4	SA
		Sucralose	56038-13-2	SA
		Sulfamethoxazole	723-46-6	SA
	CAAM	Alachlor ESA	142363–53–9	CS
		Alachlor OA	171262-17-2	CS
		Metolachlor ESA	171118–09–5	CS
		Metolachlor OA	152019–73–3	CS
Extracted surrogate	HWI	Benzoylecgonine-d3	115732-68-8	SA
Injection Internal Standards	HWI	Acesulfame-D4	1623054-53-4	TRC
		Carbamazepine-D10	132183-78-9	SA
		Sucralose-D6	1459161-55-7	TRC
		Sulfamethoxazole-D4	1020719-86-1	TRC
	CAAM	Butachlor ESA	1173022-75-7	CS
HWI = Human waste indicators (pharmaceuticals, personal care products, artificial sweeteners)<br>CAAM = Chloroacetanilide metabolites; ESA = ethane sulfonic acid; OA = oxanilic acid<br>Source column abbreviations: TRC = Toronto Research Chemicals; SA = Sigma Aldrich; CS = ChemService				

1.4.2 Aqueous extraction method for human waste indicators

A 450 mL aliquot of sample was treated with 50 mL of EDTA (80 g L^{-1}), 300 µL sulfuric acid (1:1), and 125 µL benzoylecgonine-d3 (surrogate standard). Of this mixture, 100 mL was passed through a hydrophilic-lipophilic balanced cartridge (Waters Oasis HLB 6 cc, 200 mg) using a Dionex Autotrace solid-phase extraction (SPE) unit. Prior to loading the sample, cartridges were conditioned with 5.0 mL of methanol then 5.0 mL ultra-pure water at 5.0 mL min^{-1}. After loading, cartridges were dried for 15 minutes under nitrogen gas then eluted with 5.0 mL methanol at 5.0 mL min^{-1}. Eluents were transferred to a Turbovap concentration station and dried with nitrogen gas at 50°C. After drying completely, 500 µL of methanol was used to rinse down the sides of the tubes, then extracts were brought to dryness again. Tubes were removed from the Turbovap and 50 µL of injection internal standard mix was added. This final extract was brought to a volume of 500 µL with 15 mM acetic acid in ultra-pure water and transferred to vials for analysis by LC/MS.

1.4.3 Aqueous extraction method for chloroacetanilide metabolites

This method is based upon USGS Open File Report 00-182[8]. A Dionex Autotrace Solid Phase Extraction (SPE) system fitted with a C18 cartridge (Waters SepPak C18 6cc, 500 mg). Cartridges were conditioned with 3 mL of each: methanol, ethyl acetate, methanol again, then ultra-pure water. After conditioning, the syringe was rinsed with 5 mL ethyl acetate, then 125 mL of sample was loaded. The cartridge was dried with nitrogen for 0.5 minutes, then eluted with ethyl acetate to remove the more non-polar compounds, followed by methanol to remove the more polar chloroacetanilide herbicide metabolites. Both fractions were collected in 5 mL centrifuge tubes. The ethyl acetate fraction was discarded. The methanol fraction was transferred to a Turbovap concentration station and dried with nitrogen gas at 50°C. After drying completely, 500 µL of methanol was used to rinse down the sides of the tubes, then extracts were brought to dryness again. Tubes were removed from the Turbovap and 50 µL of Butachlor ESA injection internal standard mix was added. This final extract was brought to a volume of 500 µL with 15 mM acetic acid in ultra-pure water and transferred to vials for analysis by LC/MS.

1.4.4 LC/MS/MS method for human waste indicators

Analysis was performed on an Agilent 1200 series high performance liquid chromatograph coupled to an Agilent 6430 triple quadrupole mass spectrometer with an electrospray ionization source (ESI-LC/MS/MS) following a pre-programmed gradient (below) with an additional two minutes of post run time. The injection internal standard recovery was used to adjust target analyte concentrations.

Analytical column: Zorbax Eclipse XDB-C18 column, 4.6 × 50 mm; 1.8 µ (Scheurer et. al., 2009)

Guard column: Zorbax Eclipse Plus-C18, 2.1 x 12.5 mm; 5 µ

Injection volume: 20 µL

Flow Rate: 0.5 mL minute^{-1}

Mobile phase: (A) 15 mM acetic acid in ultra-pure water; (B) 15 mM acetic acid in methanol

Column temperature: 50°C

Gradient:	Time (minutes)	% mobile phase B
	0	10
	5.0	45
	6.5	95
	15.0	95
	16.0	10

1.4.5 LC/MS/MS method for chloroacetanilide metabolites

Analysis was performed on an Agilent 1200 series high performance liquid chromatograph coupled to an Agilent 6430 triple quadrupole mass spectrometer with an electrospray ionization source (ESI-LC/MS/MS) following a pre-programmed gradient (below) with an additional two minutes of post run time. The injection internal standard recovery was used to adjust target analyte concentrations.

Analytical column: Thermo Betasil C-18, 150 x 2.1 mm; 3 µ

Injection volume: 10 µL

Flow Rate: 0.5 mL minute^{-1}

Mobile phase: (A) 15 mM acetic acid in ultra-pure water; (B) 15 mM acetic acid in methanol

Column temperature: 70°C

Gradient:	Time (minutes)	% mobile phase B
	0	20
	10.0	85
	20.0	85
	22.0	20

1.4.6 Quality control – non-PFAS organics

Ongoing quality control included analysis of laboratory reagent blanks (ultra-pure water extracted by SPE – for pharmaceuticals, personal products, and artificial sweeteners, the blank was prepared in the same

manner as samples, with the addition of EDTA, sulfuric acid, and surrogate standard). Laboratory fortified blanks (ultra-pure water) and laboratory matrix spikes (study samples) were prepared similar to samples, with the addition of a target analyte mixture. Laboratory reagent blanks, fortified blanks, and matrix spikes were performed for every 20 samples. Field duplicates and blanks were also collected and analyzed. Internal calibration was utilized. Calibration checks and blanks were performed every 10 samples.

1.5 Inorganics and total organic carbon

Inorganics analysis was conducted at WEAL. The elemental forms of major cations, metals, sulfur and phosphorous were analyzed using EPA Method 200.7, rev. 4.4. The elements analyzed were As, Ca, Cu, Fe, K, Pb, Mg, Mn, Na, P, S and Zn. Total ammonia/ammonium nitrogen (NHx), nitrate-nitrogen plus nitrite nitrogen (NOx) and chloride were analyzed using *Standard Methods*[9] 4500-NH3 H, 4500-NO3 F and 4500-Cl G, respectively. Laboratory pH, conductivity and alkalinity were analyzed following *Standard Methods*[9] 4500H+ B, 2510B and 2320B. Non-purgeable total organic carbon (TOC) was analyzed following *Standard Methods* 5310B-2000 TOC[9]. TOC was chosen, rather than dissolved organic carbon (i.e., TOC of filtered samples), because of the possibility of colloidal or particulate organic matter (which could transport PFAS) content in karst and fractured rock groundwater environments. Standards and reagents are shown in Table S13 and Table S14.

Table S13. Standards and reagents for inorganics analyzed with ICP-OES, alkalinity and total organic carbon

Parameter(s)	Standards	Reagents
Arsenic, calcium, iron, magnesium, manganese, phosphorus, potassium, sodium, sulfur, zinc	Inorganic Ventures: Multi-element custom grade solution in 5% nitric acid	Fisher Chemical: Nitric acid, Optima grade. Nitric acid, Trace metal grade.<br>Reagent water: ASTM Type I, 18.2 MΩ<br>Inorganic Ventures: Single element standards. Yttrium (internal standard), Cerium (interference correction), Cobalt (interference correction)

Parameter(s)	Standards	Reagents
		Alfa Aesar: Cesium nitrate, metals basis 99.99% purity
Alkalinity (as CaCO3), pH, & conductivity	Fisher Scientific: Sodium bicarbonate, NaHCO3, anhydrous, Certified ACS. Potassium chloride, Certified ACS grade.<br><br>LabChem: Buffer solutions, pH 4.0, 7.0, and 10.0.	Fisher Chemical: Sulfuric acid, 95-98%, ACS grade. Potassium chloride, Certified ACS grade.<br><br>Reagent water: ASTM Type I, 18.2 MΩ
Total organic carbon	Acros Organics: Potassium hydrogen phthalate, C8H5KO4, Primary grade standard<br><br>Fisher Scientific: Sodium bicarbonate, NaHCO3, anhydrous, Certified ACS<br><br>Fisher Scientific: Sodium carbonate, Na2CO3, anhydrous, Certified ACS	Fisher Chemical: Sulfuric acid, Trace metal grade. Phosphoric acid, 85%, Certified ACS<br><br>Reagent water: ASTM Type I, 18.2 MΩ

Table S14. Reagents (R) and standards (Std) for nitrate plus nitrite, chloride and ammonium

Analyte	Chemical	CAS	Source	Grade	Reagent/Std
NO_3^- + NO_2^- (N)	Ammonium chloride	12125-02-9	Sigma	99.5%	R
NO_3^- + NO_2^- (N)	Ethylenediamine tetraacetic acid disodium salt dihydrate (EDTA)	6381-92-6	Fisher	ACS	R
NO_3^- + NO_2^- (N)	Sodium hydroxide	1310-73-2	Fisher	ACS	R
NO_3^- + NO_2^- (N)	Sulfanilamide	63-74-1	Sigma	>98.0%	R

Analyte	Chemical	CAS	Source	Grade	Reagent/Std
NO_3^- + NO_2^- (N)	N-(1-Naphthyl) ethylenediamine, dihydrochloride (NED)	1465-25-4	Acros	98.0+%	R
NO_3^- + NO_2^- (N)	85% phosphoric acid	7664-38-2	Fisher	ACS	R
NO_3^- + NO_2^- (N)	Sodium nitrite	7632-00-0	Fisher	ACS	Std
NO_3^- + NO_2^- (N)	Potassium nitrate	7757-79-1	Sigma	99.0%	Std

Analyte	Chemical	CAS	Source	Grade	Reagent/Std
Chloride	Mercuric thiocyanate	592-85-8	Sigma	96.5%-103.5%	R
Chloride	Methanol	67-56-1	Fisher	ACS	R
Chloride	Iron (III) nitrate nonahydrate	7782-61-8	Sigma	>98.0%	R
Chloride	Nitric acid	7697-37-2	Fisher	ACS+	R
Chloride	Sodium chloride	7647-14-5	Fisher	ACS	Std

Analyte	Chemical	CAS	Source	Grade	Reagent/Std
NH_4 as NH_3-N	Ammonium chloride	12125-02-9	Sigma	99.5%	Std
NH_4 as NH_3-N	Crystalline phenol	108-95-2	Sigma	ACS	R
NH_4 as NH_3-N	Sodium hydroxide	1310-73-2	Fisher	ACS	R
NH_4 as NH_3-N	8.25% Bleach (Sodium hypochlorite)		Clorox		R
NH_4 as NH_3-N	Ethylenediamine tetraacetic acid disodium salt dihydrate (EDTA)	6381-92-6	Fisher	ACS	R
NH_4 as NH_3-N	Sodium Nitroferricyanide	13755-38-9	Fisher	ACS	R
NH_4 as NH_3-N	Sulfuric acid	7664-93-9	Fisher	TMG	R

The elemental forms of major cations, metals, sulfur and phosphorous were analyzed using an Agilent Technologies 700 Series inductively coupled plasma optical emission spectrometer by EPA Method 200.7,

rev. 4.4. Total ammonia/ammonium nitrogen (NHx), nitrate-nitrogen plus nitrite nitrogen (NOx) and chloride were analyzed using a Lachat 8500 flow-injected autoanalyzer by Standard Methods[9] 4500-NH3 H, 4500-NO3 F and 4500-Cl G, respectively. Laboratory pH, conductivity and alkalinity were analyzed using a Mantech AutoMax 73 auto-titration system following Standard Methods 4500H+ B, 2510B and 2320B. Non-purgeable TOC was analyzed by combustion using a Shimadzu TOC Analyzer following Standard Methods 5310B-2000 TOC.

MDLs for all non-PFAS compounds/parameters are shown in Table S15.

Table S15. Method detection limits (MDLs) for non-PFAS parameters

Parameter(s)	Method detection limit ($\mu g\ L^{-1}$)
CAAMs (all four analytes)	0.08
Acesulfame	0.005
Sucralose	0.025
Carbamazepine	0.002
Sulfamethoxazole	0.005
Arsenic	4
Calcium	51
Iron	13
Magnesium	4
Manganese	1
Phosphorus	11
Potassium	16
Sodium	246
Sulfur	190
Zinc	1
Chloride	500
Ammonia/ammonium (as N)	10
Nitrate plus nitrite (as N)	100
Alkalinity (as CaCO3)	400
Total organic carbon	300

1.6 Land use data analysis

The Wiscland2[10] spatial dataset was used for land use analysis. Table S16 provides land use definitions, precision and accuracy from the User Guide. The Wiscland2 user guide[10] can be accessed, starting from the web page https://dnr.wisconsin.gov/maps/WISCLAND, by clicking the link to the GIS Open Data Portal (currently accessible at https://data-wi-dnr.opendata.arcgis.com/) and then searching for "Wiscland2" in the search box.

Table S16. Wiscland2*[10]* definitions of land use types used in this study

Land use category	Definition# from Wiscland2 User Guide	Additional notes	Precision*	Accuracy*
Developed	Structures and areas associated with intensive human activity and land use. Note: Areas meeting the requirements of both Urban/Developed and Forest, Wetland, or Grassland classes should be classified in the Urban/Developed category (e.g., residential areas with 75% crown closure of trees but 25% impervious cover would be classified as Urban/Developed, rather than Forest).	Since most Wisconsin cities have municipal water supplies, private wells are not typically found in "urban" areas. Study wells in this land use category predominantly came from areas that are best described as "developed".	91%	0.99
Agricultural	Land under cultivation for food or fiber.	Includes the following categories: Cash Grain, Continuous Corn, Dairy Rotation, and Potato/Vegetable.	NA	NA
Grassland	Lands covered by non-cultivated herbaceous vegetation predominated by perennial grasses. Forbs and other grass-like plants	Includes land used for livestock forage production and grazing.	84%	0.95

Land use category	Definition# from Wiscland2 User Guide	Additional notes	Precision*	Accuracy*
	may be present or sometimes even dominant.			
Forested	An upland area of land covered with woody perennial plants, the trees reaching a mature height of at least 6 feet tall with definite crown (closure of at least 10%). Note: If an area meets the requirements of Forested Wetland, it should take precedence over any other Forest Category.		78%	0.96

Notes:

#From Appendix C of the Wiscland2 User Guide[10]

*From Table 2 of the Wiscland2 User Guide[10]

NA: Not available

2.0 Results and discussion

2.1 Occurrence

Figure S2 displays boxplots of PFAS concentrations with a minimum comparison level of 0.18 ng/L and includes modeled concentrations of three compounds (PFBA, PFDoA and PFBS) above that minimum comparison level but below their respective detection limits (0.327, 0.256 and 0.219 ng/L, respectively). Concentrations were modeled using regression on order statistics[11,12]. Figure S3 shows plots of those regression models and the detected and modeled concentrations of PFBA, PFDoA and PFBS.

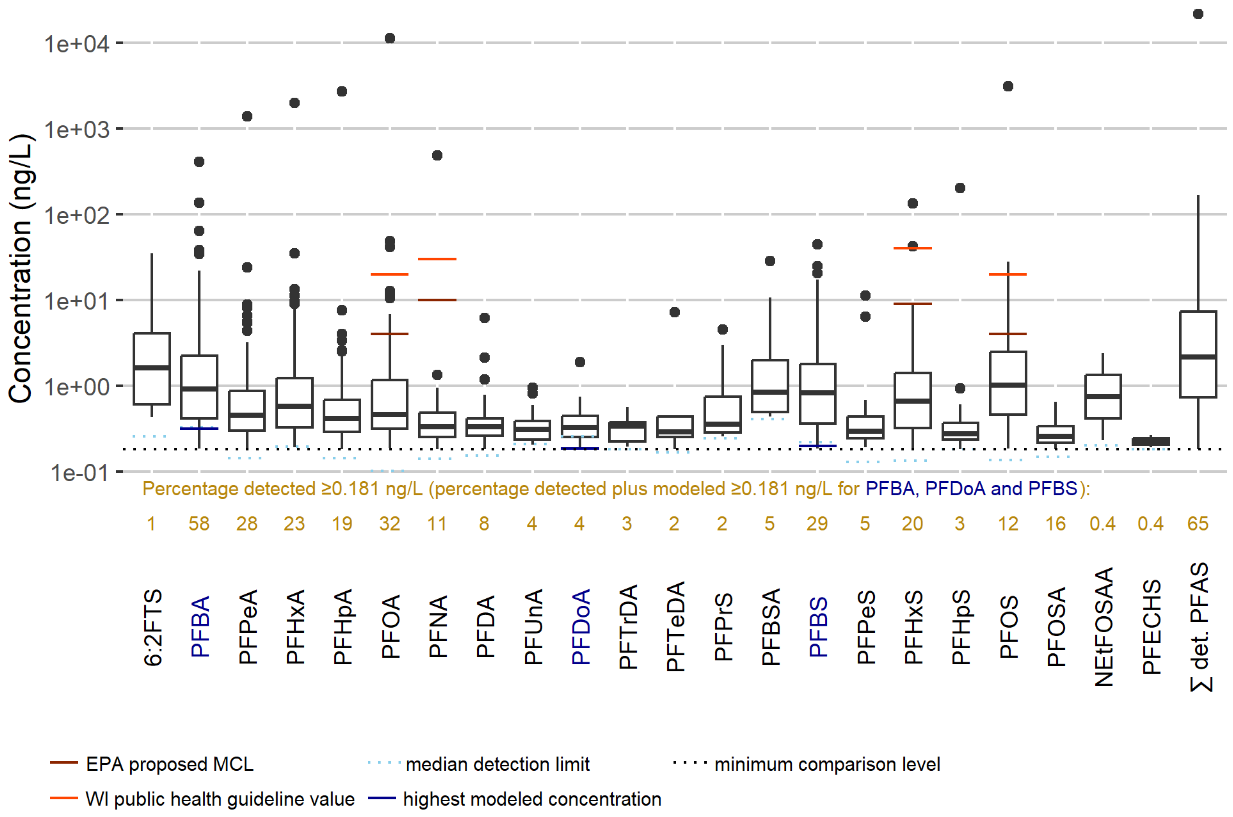

Figure S2. Boxplots of detected and modeled PFAS concentrations at or above 0.181 ng/L

The following compounds were analyzed but not detected in any of the 450 samples (CAS number for each compound provided in parentheses):

- The perfluoroalkyl sulfonic acids PFNS (68259-12-1), PFDS (335-77-3) and PFDoS (79780-39-5)
- The PFHxS precursor PFHxSA (41997-13-1) and the PFOS precursors NMeFOSA (31506-32-8), NMeFOSE (24448-09-7), NMeFOSAA (2355-31-9), NEtFOSA (4151-50-2) and NEtFOSE (1691-99-2)
- The fluorotelomer sulfonic acids 4:2FTS (757124-72-4), 8:2FTS (39108-34-4) and 10:2FTS (120226-60-0)
- The fluorotelomer carboxylic acids 3:3FTCA (356-02-5), 5:3FTCA (914637-49-3) and 7:3FTCA (812-70-4)
- The fluorotelomer unsaturated carboxylic acids 6:2FTUCA (70887-86-6), 8:2FTUCA (70887-84-2) and 10:2FTUCA (70887-94-4)
- The ether-containing fluorosubstances HFPO-DA (13252-13-6), DONA (919005-14-4), 9Cl-PF3ONS (756426-58-1) and 11Cl-PF3OUdS (763051-92-9)

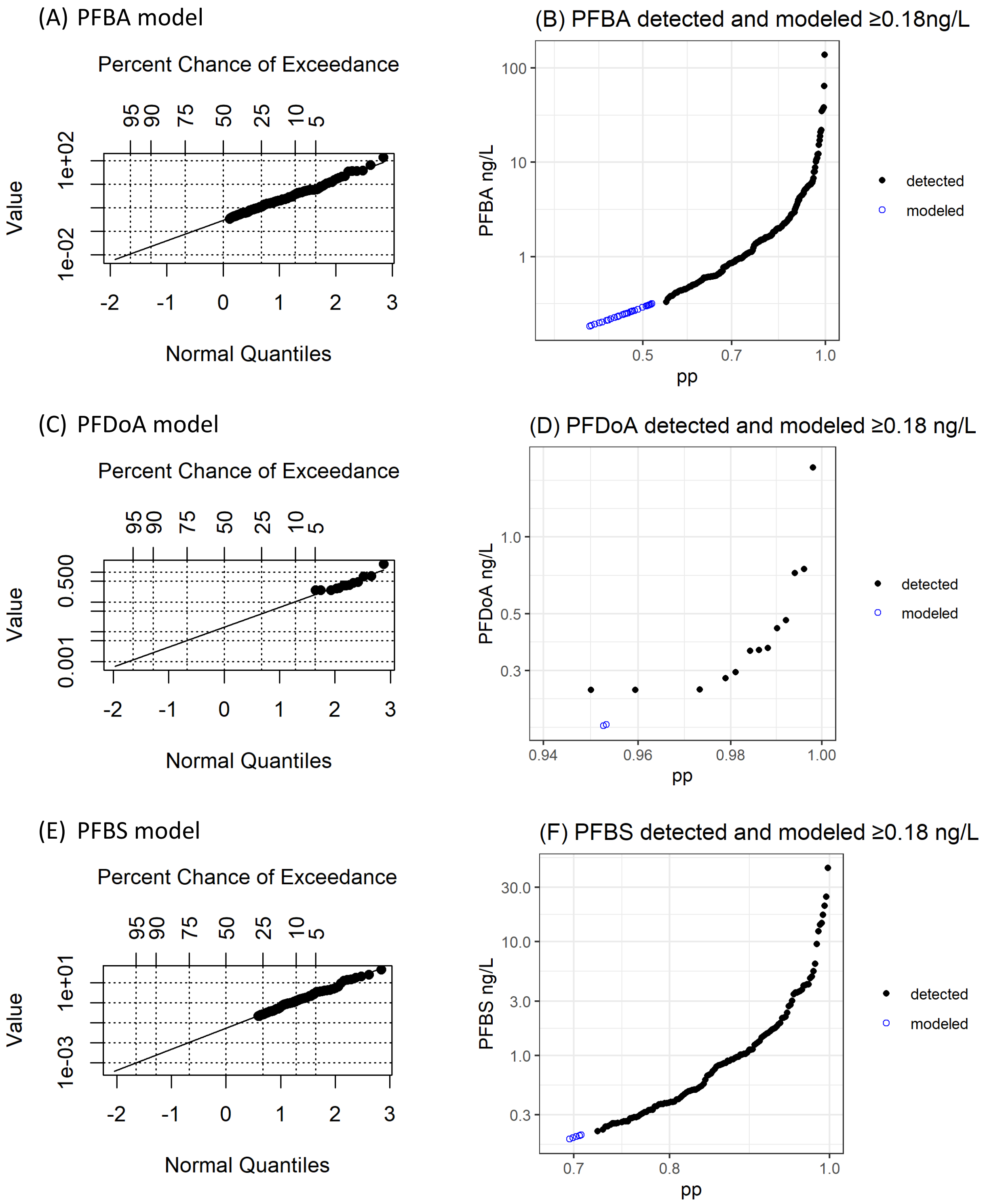

Figure S3. Regression on order statistics models for the three compounds with modeled concentrations above 0.181 ng/L. In A, C and E, lines (the regression model, note non-uniform x-axis) are fit to the observed data (points). B, D and F show the observed data (black points) and modeled concentrations (blue open circles) ≥0.181 ng/L.

Concentrations of non-PFAS lab parameters for the 450 samples are shown in Figure 4. For detection limits, see Table S15. Values of field parameters are shown in Figure S5.

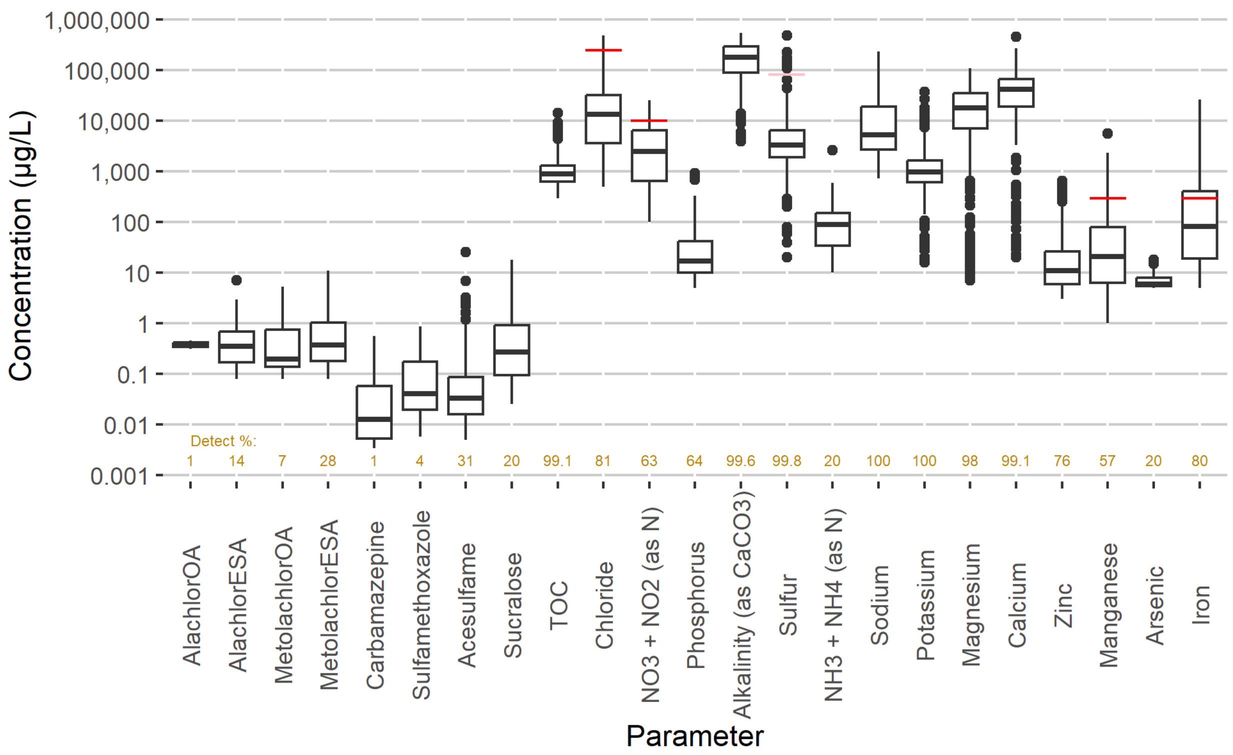

Figure S4. Prevalence of non-PFAS analytes/parameters in the 450 samples. Red lines indicate Wisconsin groundwater standards for compounds/parameters with one or more samples above. The pink line (sulfur) is the corresponding concentration of S, under the assumption that all S is sulfate, to the public welfare groundwater standard for sulfate.

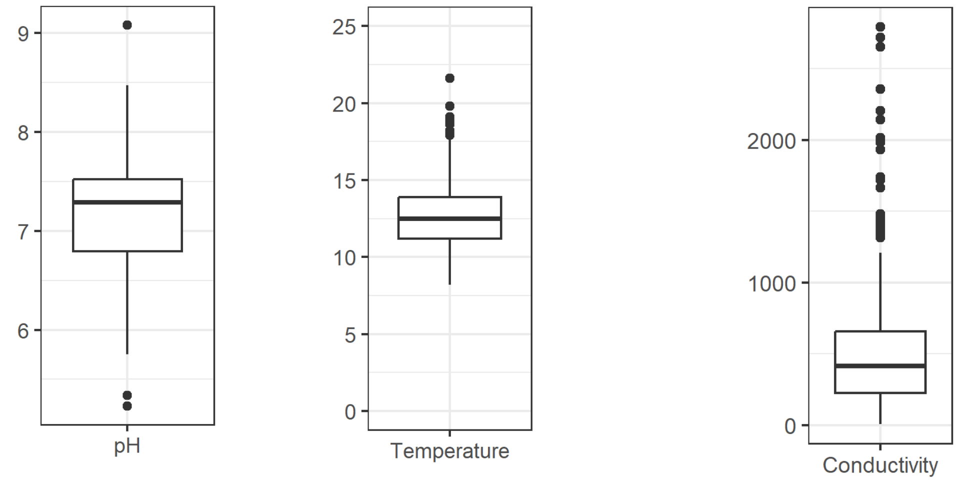

Figure S5. Field measurement values of water pH, temperature (°C) and conductivity (µS/cm) in the 450 samples.

2.2 Source tracing

A subset of project groundwater samples (collected from residences with private wells) with all PFAS detected at or below Wisconsin precipitation levels is shown in Figure S6. Wisconsin precipitation levels are considered to be the highest site median, per compound, from Table 2 of Pfotenhauer et al.[13]

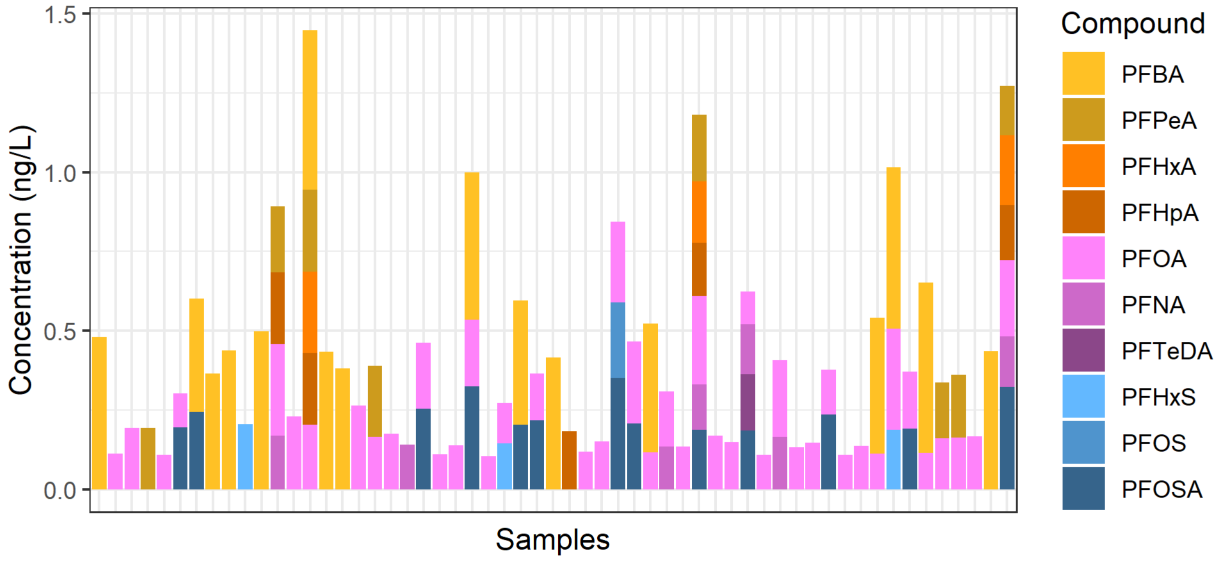

Figure S6. Project samples (n=57) with all detected PFAS below 2020 Wisconsin precipitation levels, considered to be the highest site median in Table 2 of Pfotenhauer et al.[13]

In Tables S17 and S18, proportionality testing of detection(s) of one or more PFAS by land use type is compared using two different choices for dealing with censored data. Choice A (used in the manuscript) is to count all detections, without any adjustment to the laboratory reported values. Choice B is to count a result as a detection only if the detected concentration was ≥0.409 ng/L (the highest LOD for all 22 PFAS detected in the study).

Table S17. Comparison of proportionality using either (A) detection(s) at any level or (B) detection(s) ≥0.409 ng/L

Land use type	Number of samples	(A) Detection(s) at any concentration	(B) Detection(s) ≥0.481 ng/L only	(A) Detection rate at any concentration	(B) Detection rate ≥0.409 ng/L
Forested	246	173	122	70%	51%
Agricultural	110	71	58	65%	54%
Grassland	49	34	25	69%	53%
Developed	45	40	36	89%	87%

Table S18. Proportionality test outcomes using (A) detection(s) at any level or (B) detection(s) ≥0.409 ng/L

Comparison	(A) p-value for detection(s) at any concentration	(B) p-value for detection(s) ≥0.409 only
Agricultural versus forested	0.34	0.76
Agricultural versus grassland	0.68	1
Agricultural versus developed	**0.004**	**0.0002**
Forested versus grassland	0.16	0.94
Forested versus developed	**0.02**	**0.00002**
Grassland versus developed	**0.04**	**0.001**

Figure S7 shows the rate of detection of individual PFAS, at any concentration, for different land use categories. Proportionality test (R function ‘prop.test’) p-values less than 0.05 (bolded in Table S19) indicate which comparisons have significant differences in detection rate. Differences in PFOA

concentrations in the agricultural to developed comparison are moderately significant, with a p-value of 0.056.

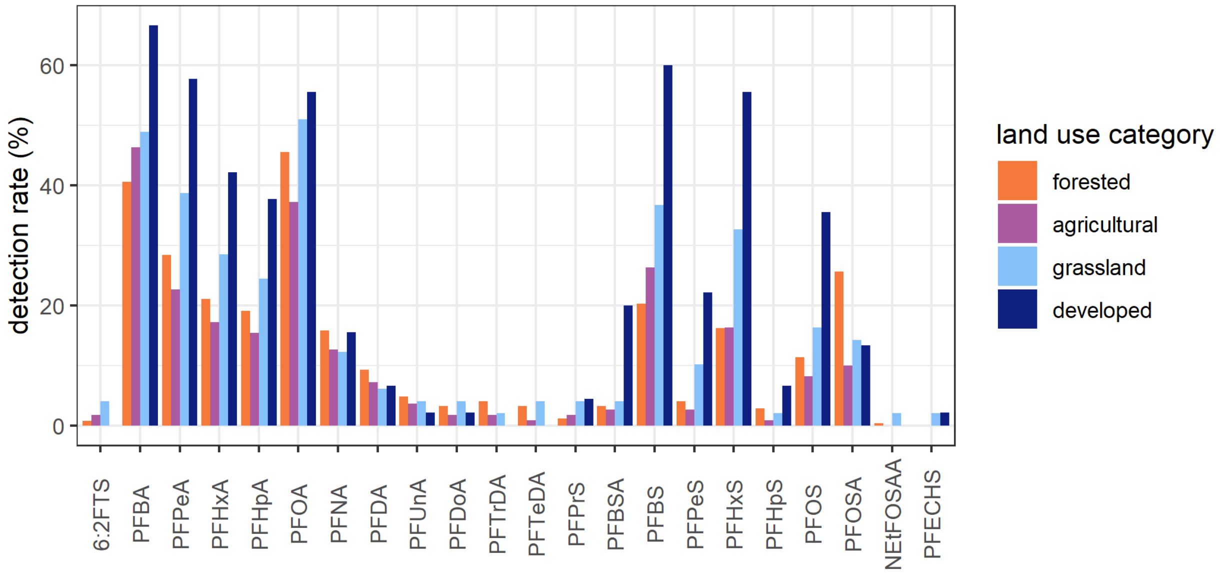

Figure S7. Rate of detection of individual PFAS, at any concentration, across land use categories.

Table S19. Significance (p-values) of detection rates across land use categories. P-values < 0.05 indicate comparisons where the detection rate is significantly different between the two categories (for which is higher, see Figure S4).

	forested to agricultural	forested to grassland	forested to developed	agricultural to grassland	agricultural to developed	grassland to developed
PFBA	0.38	0.36	**0.002**	0.89	**0.03**	0.13
PFPeA	0.31	0.21	**0.0002**	0.06	**0.00006**	0.1
PFHxA	0.48	0.34	**0.004**	0.16	**0.002**	0.24
PFHpA	0.5	0.51	**0.01**	0.25	**0.005**	0.24
PFOA	0.18	0.58	0.28	0.15	0.056	0.81
PFNA	0.54	0.67	1	1	0.83	0.87
PFDA	0.66	0.65	0.77	1	1	1
PFUnA	0.81	1	0.69	1	1	1
PFDoA	0.68	1	1	0.77	1	1
PFTrDA	0.44	0.79	0.35	1	0.9	1
PFTeDA	0.35	1	0.46	0.47	1	0.51
PFPrS	1	0.41	0.36	0.77	0.71	1
PFBSA	1	1	**0.00005**	1	**0.0009**	**0.04**
PFBS	0.26	**0.02**	**0.00000008**	0.26	**0.0002**	**0.04**
PFPeS	0.75	0.15	**0.00004**	0.11	**0.0003**	0.19
PFHxS	1	**0.01**	**0.00000002**	**0.04**	**0.000002**	**0.04**
PFHpS	0.45	1	0.4	1	0.14	0.55
PFOS	0.47	0.47	**0.00008**	0.21	**0.00007**	0.058
PFOSA	**0.001**	0.13	0.11	0.61	0.75	1

An expanded version of Figure 4 of the manuscript (Spearman correlations), showing additional study variables, is shown as Figure S8.

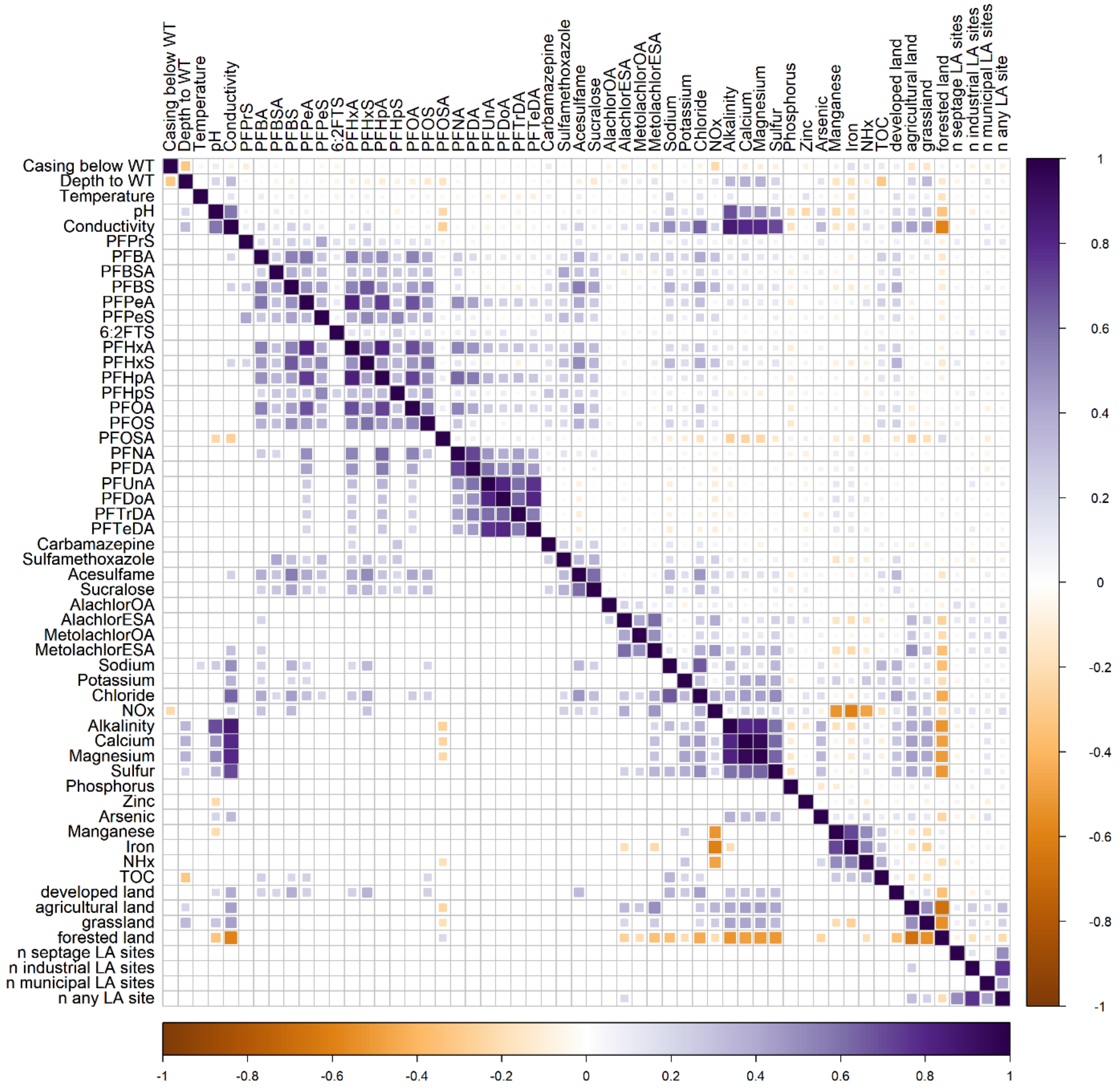

Figure S8. Correlation plot with intensity and amount of color indicating the value of the Spearman correlation coefficient (ρ) between two variables. The upper-right triangle shows all correlation values while the lower-left triangle shows colored squares only for significant correlations (Holm sequentially adjusted $p < 0.05$).

Figure S9 displays concentrations of PFBA, which are higher in a portion of western Wisconsin compared to the rest of the state (Mann-Whitney-Wilcoxon rank sum test $p = 8*10^{-12}$).

The single highest PFBA concentration (largest diameter circle on Figure S9, not located in the yellow-shaded portion of western Wisconsin) occurred at the same site as the PFOA concentration of 11,300 ng/L (an unconsolidated aquifer in north-central Wisconsin). In the vicinity of that original sampling site, water from additional homes with private wells has been sampled for PFAS as part of a separate effort from this study. Sampling efforts in the area are ongoing and information is available at https://dnr.wisconsin.gov/topic/PFAS/Stella.html. That web page is currently planned to be updated as additional information emerges.

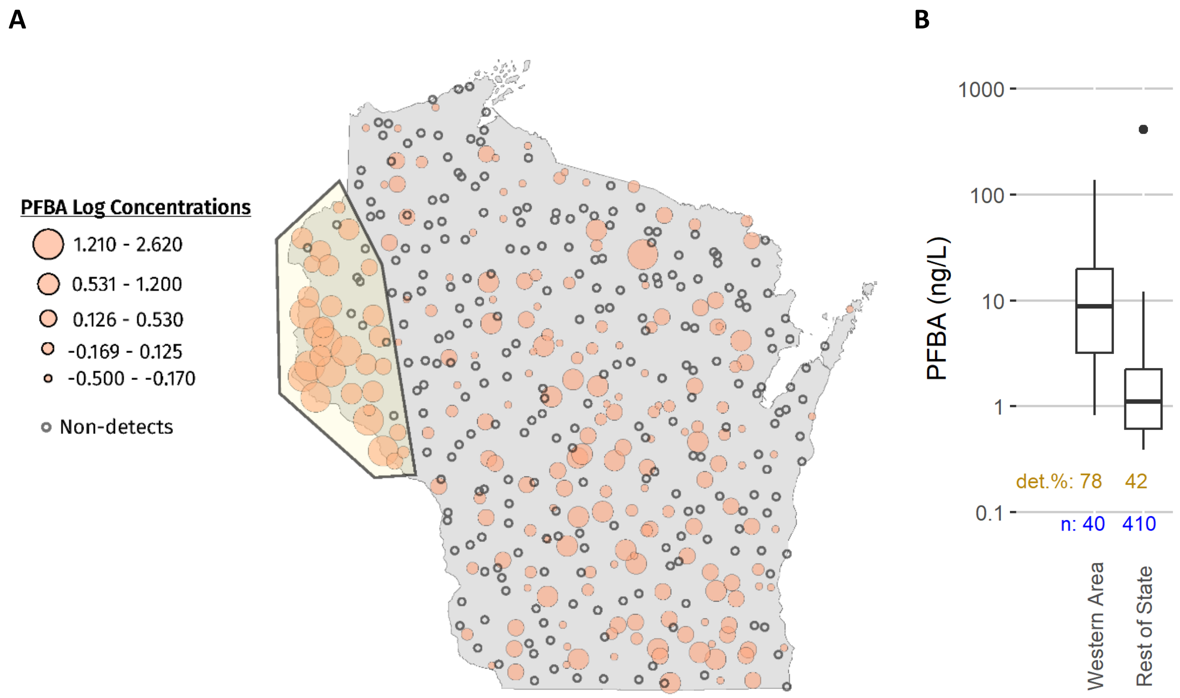

Figure S9. A) Log of concentrations (ng/L) of PFBA at the 450 project sites (Reproduced with permission from the Wisconsin Department of Natural Resources) and B) comparison of concentrations in the Western Area (shaded yellow on *A*) vs. the rest of the state.

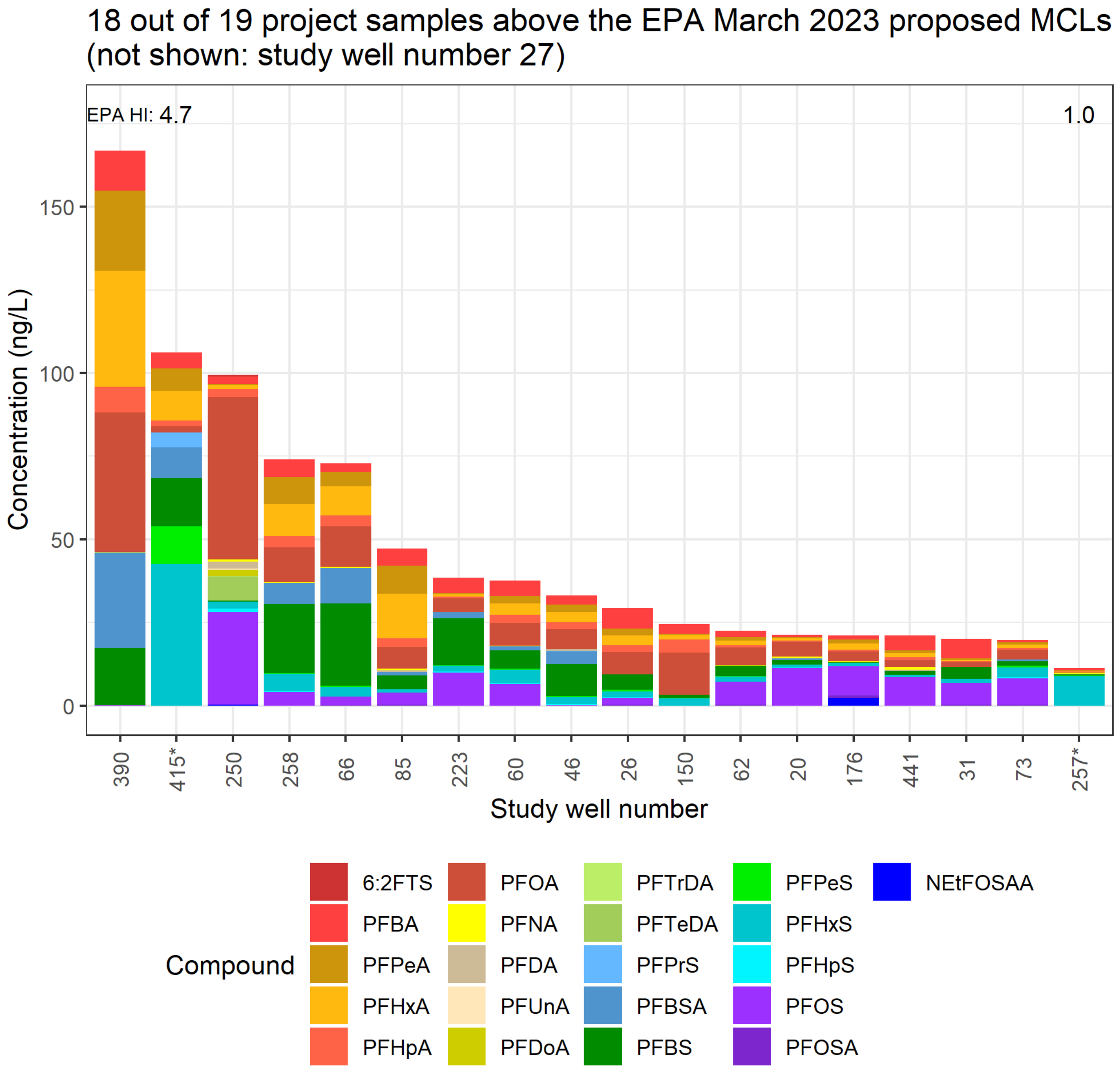

*Above the EPA March 2023 proposed Hazard Index (HI) only, PFOA and PFOS both less than 4 ng/L

Figure S10. Stacked column plots of the PFAS detected in 18 of the 19 samples with one or more PFAS above the EPA March 2023 proposed MCLs.

Figure S10 allows comparison of PFAS signatures (combinations and ratios of compounds detected) in the samples shown. Signatures may be affected by source type as well as transport in soil and groundwater. The sample not shown is the one with PFOA detected at 11,300 ng/L; complete results for all project samples can be found in Table S22.

Tables S20 through S24 are provided in an accompanying Microsoft Excel spreadsheet. The p-values listed in Table S24 were calculated with the R package “psych”, version 2.3.6, using:

psych::corr.test(data, use="complete", method = "spearman", adjust="holm", alpha = 0.05).

Table S20. Locations of project wells and number of land application sites within 1000 m

Table S21. Field parameter data with laboratory pH and conductivity results

Table S22. Sample analytical data for primary samples

Table S23. Sample analytical data for field quality control samples (field blanks and duplicates)

Table S24. Significance (Holm sequentially adjusted p-values) of the correlations in Figure 4 of the manuscript.

References

(1) Michigan Department of Environmental Quality. *General PFAS Sampling Guidance*; 2018. https://www.michigan.gov/pfasresponse/-/media/Project/Websites/PFAS-Response/Sampling-Guidance/General.pdf?rev=5fb24f7dabf0468b9415679b60681503 (accessed 2021-03-08).

(2) U.S. Environmental Protection Agency. *Other Test Method 45 (OTM-45) Measurement of Selected Per- and Polyfluorinated Alkyl Substances from Stationary Sources*. https://www.epa.gov/emc/emc-other-test-methods (accessed 2023-02-11).

(3) U.S. Environmental Protection Agency. *Draft Method 1633: Analysis of Per- and Polyfluoroalkyl Substances (PFAS) in Aqueous, Solid, Biosolids, and Tissue Samples by LC-MS/MS*. https://www.epa.gov/system/files/documents/2021-09/method_1633_draft_aug-2021.pdf (accessed 2023-02-11).

(4) Barzen-Hanson, K. A.; Field, J. A. Discovery and Implications of C_2 and C_3 Perfluoroalkyl Sulfonates in Aqueous Film-Forming Foams and Groundwater. *Environ. Sci. Technol. Lett.* **2015**, *2* (4), 95–99. https://doi.org/10.1021/acs.estlett.5b00049.

(5) Meng, P.; DeStefano, N. J.; Knappe, D. R. U. Extraction and Matrix Cleanup Method for Analyzing Novel Per- and Polyfluoroalkyl Ether Acids and Other Per- and Polyfluoroalkyl Substances in Fruits and Vegetables. *J. Agric. Food Chem.* **2022**, acs.jafc.1c07665. https://doi.org/10.1021/acs.jafc.1c07665.

(6) U.S. Environmental Protection Agency. Method 533: Determination of Per- and Polyfluoroalkyl Substances in Drinking Water by Isotope Dilution Anion Exchange Solid Phase Extraction and Liquid Chromatography/Tandem Mass Spectrometry, 2019. https://www.epa.gov/sites/default/files/2019-12/documents/method-533-815b19020.pdf (accessed 2023-09-30).

(7) U.S. Environmental Protection Agency. CompTox Chemicals Dashboard. https://comptox.epa.gov/dashboard/ (accessed 2023-03-19).

(8) Zimmerman, L. R.; Hostetler, K. A.; Thurman, E. M. *Methods of Analysis by the U.S. Geological Survey Organic Geochemistry Research Group-Determination of Chloroacetanilide Herbicide Metabolites in Water Using High-Performance Liquid Chromatography-Diode Array Detection and High-Performance Liquid Chromatography/Mass Spectrometry*; U.S.G.S. Open-File Report 00–182; 2000. https://pubs.usgs.gov/of/2000/0182/report.pdf.

(9) Bridgewater, L. *Standard Methods for the Examination of Water and Wastewater*, 22nd ed.; American Public Health Association, 2012.

(10) Wisconsin Department of Natural Resources - GIS Services Section. *Wiscland 2 Land Cover User Guide*; Madison, WI, USA, 2016. https://dnr.wisconsin.gov/maps/WISCLAND (accessed 2023-03-17).

(11) Helsel, D. R. *Statistics for Censored Environmental Data Using Minitab and R*, 2nd edition.; Wiley Series in Statistics in Practice; Wiley: Denver, 2012.

(12) Lee, L. *NADA (version 1.6-1.1) Nondetects and Data Analysis for Environmental Data*. https://www.rdocumentation.org/packages/NADA/versions/1.6-1.1 (accessed 2023-06-22).

(13) Pfotenhauer, D.; Sellers, E.; Olson, M.; Praedel, K.; Shafer, M. PFAS Concentrations and Deposition in Precipitation: An Intensive 5-Month Study at National Atmospheric Deposition Program – National Trends Sites (NADP-NTN) across Wisconsin, USA. *Atmospheric Environment* **2022**, *291*, 119368. https://doi.org/10.1016/j.atmosenv.2022.119368.
